# The devil’s in the detail: an appraisal of the use of innovative financing mechanisms for pandemic prevention, preparedness and response

**DOI:** 10.1186/s12992-025-01103-w

**Published:** 2025-03-27

**Authors:** Blagovesta Tacheva, Garrett Wallace Brown, David Bell, Jean von Agris

**Affiliations:** 1 School of Politics and International Studies (POLIS), University of Leeds, Leeds, LS2 9JT UK; 2 Independent Consultant, Lake Jackson, TX USA

**Keywords:** Innovative financing mechanisms, Financing pandemic preparedness, Pandemic preparedness and response, PPR, PPPR, Global health financing

## Abstract

This is the first published study examining whether, and to what degree, innovative financing could effectively support the financing needs of the global pandemic prevention, preparedness and response (PPPR) agenda. **Background: What is already known?** In the context of global health, innovative financing encompasses a range of financial instruments that supplement international development assistance and other traditional sources of financing, with the intention of mobilising additional resources and channelling them more effectively. Examples including Advance Market Commitments (AMCs), Advance Purchase Commitments (APCs), vaccine bonds and pandemic bonds, have been used in the past to address major disease outbreaks, such as the Ebola and Covid-19 crises. Following the Covid-19 outbreak, innovative financing has been proposed as a major vehicle to fund PPPR. **Results: What are the new findings?** Despite recent pronouncements that innovative financing has ‘huge untapped potential’ for PPPR, there is little evidence within the literature to support such claims. This has been confirmed by our examination of four innovative financing mechanisms and their historical use in response to disease outbreaks. Our findings suggest that flaws and trade-offs in the design and application of these mechanisms have resulted in failure to deliver on their promise, raising concerns regarding their prospective use in financing PPPR. Although innovative financing could play a role, existing mechanisms in health have not generated the scale of funds proposed. In addition, the amounts generated have historically focused on specific interventions, which threaten to enhance fragmentation (disjointed financing of health) and alignment failures (not well integrated within overall national strategic plans) with and within PPPR. **Conclusions: What do the new findings imply?** Our findings reveal a set of innovative financing tools shrouded in unsubstantiated claims to success and effectiveness that look to have underwhelming promise of ‘value for money’ in global health. This stems from evidence suggesting design flaws, inadequate application, lack of transparency, private sector profiteering and associated opportunity costs. Thus, contrary to popular claims, they may not be the ‘silver bullet’ for bridging PPPR financing gaps and addressing costly, complex and multifaceted PPPR interventions.

## Background

The World Bank and World Health Organization (WHO) have provided estimates of the annual pandemic preparedness and response financing needs in a report produced for the G20 Joint Finance and Health Task Force. Supporters of the prevailing pandemic prevention, preparedness and response (PPPR) agenda are now coalescing around these new figures as the basis for future PPPR needs. The World Bank and WHO estimate that low- and lower-middle income country (LMIC) governments and donors need to invest US$31.1 billion annually in PPPR, of which US$26.4 billion needs to be invested by LMICs and US$4.7 billion at the international level. The report also acknowledges that LMIC countries are unlikely to meet their national PPPR financing requirements, estimating that there is an overall annual funding gap of US$10.5 billion to be addressed by new overseas development assistance (ODA) [1]. This represents a significant investment, especially when compared to other global health requirements, while bearing in mind the current WHO budget is roughly US$3.8 billion a year. The challenge of meeting these costs is not insignificant. The economic repercussions of the economic impact of Covid-19, the war in Ukraine and ‘donor fatigue’ pose major challenges in mobilising the new ODA requirements. In addition, the World Bank-administered Pandemic Fund, currently the main instrument for financing PPPR, has only raised US$1.85 billion to date [2]. When the Pandemic Fund issued its first call for applications to request financing for PPPR implementation activities in May 2023, the scheme quickly became eight times oversubscribed [3]. The goal of meeting this challenge has given impetus to search for financing alternatives.

In this context, ‘innovative financing’ mechanisms have been championed as a possible solution to close the estimated US$10.5 billion gap in international assistance for PPPR. The World Economic Forum (WEF) argues that innovative financing can help prepare for future pandemics, citing its “huge untapped potential… to prevent outbreaks long before they reach epidemic or pandemic proportions” and save lives by making the rapid deployment of health interventions possible through the swift and efficient use of funds [4]. Moreover, the WHO Secretariat for the Pandemic Agreement has cited innovative financing as a key component for the Coordinating Financial Mechanism proposed under Article 20 (PPPR financing) of the latest draft of the Pandemic Agreement [5]. This same mechanism will also channel financing for the revised International Health Regulations, thus increasing the potential role of innovative financing for emergency preparedness more broadly [6].

Innovative financing refers to an array of financial solutions and mechanisms, “that can be used to bridge the gap between traditional forms of development funding and the financing required to achieve development goals” [7]. We adopt a broad definition that encompasses two distinct dimensions of innovative financing approaches: 1) to mobilise resources, including through private investors, that complement existing financial flows from ODA and philanthropies; and 2) to increase the efficiency and effectiveness of financial flows that address global health challenges [8]. In line with this understanding, advocates suggest that innovative financing promises to not only bring much needed additional sources of funding for global health and PPPR through a broad range of approaches, but also to “make spending much, much more effective; that we get more bang for the buck” [7, 9].

Innovative financing as a way to provide supplementary financing for global health rose to prominence in 2002, following the International Conference on Financing for Development in Monterrey [10]. In 2008, the high-level Taskforce on Innovative International Financing for Health Systems was established with the aim to “identify innovative and additional sources of funding for health systems strengthening in the 49 lowest-income countries in the world,” [11] supplementing development assistance for health in reaching the Millenium Development Goals (MDGs) and “addressing the growing non-communicable disease burden in LMICs” [10]. However, the application of innovative financing tools for PPPR can be traced back to the 2014–2016 Ebola virus outbreak in West Africa, which put into sharp relief the lack of preparedness “to provide a rapid, predictable, coordinated, and scaled-up response” to infectious disease outbreaks with pandemic potential [12]. Specifically, “it highlighted the gap between countries’ commitments for outbreak preparedness, detection, and response, as required under the International Health Regulations, and their actual ability to respond when needed [,] partly due to a lack of financing” [13]. The impetus to address this PPPR financing challenge led to the proliferation of innovative financing solutions to fill the funding gap, such as vaccine bonds, pandemic bonds, advance purchase commitments (APCs) and advance market commitments (AMCs).

Recently, “the criticality of these financing models” was brought to the fore by the Covid-19 pandemic and concomitant “debates about how to finance the research and delivery of vaccines, and how to ensure global and equitable access” [14]. In this context, the Gavi COVAX Advance Market Commitment (AMC) was launched with a stated intent to ensure equitable access to vaccines for LMICs [15]. As was argued at the 2022 World Economic Forum, such “innovative mechanisms have been used to optimize public and private sector funds and reduce financial risk, in order to accelerate access to health and medical interventions” [4]. This potential was again raised at the 2024 WEF annual Davos meeting during debates about how best to respond to Disease-X [16].

However, despite this optimism regarding the potential of innovative financing tools for PPPR, there are significant questions about their appropriateness. As part of this inquiry, “we must ask whether the costs of innovative financing mechanisms are worth it” [14]. In a bid to address these questions, we assessed four key innovative financing mechanisms for health to determine their potential suitability to respond to existing PPPR financing needs: the International Financing Facility for Immunisation (IFFIm), the Pandemic Emergency Financing Facility (PEF), the Gavi Ebola Advance Purchase Commitment (APC) and the Gavi COVAX Advance Market Commitment (AMC). In so doing, we scrutinise claims that innovative financing represents a ‘huge untapped potential’ for PPPR and whether it benefits the intended recipients. We selected these mechanisms because three of them (the IFFIm, APCs and AMCs) are the most routinely discussed as having the greatest potential for PPPR, while the PEF is examined because it was the sole innovative financing mechanism dedicated only to pandemic preparedness prior to Covid-19. Since the existing literature on these mechanisms is heavily dominated by innovative financing proponents and organisations closely associated with the mechanisms, which tend to highlight successful elements of their programmes and innovative financing in general (largely for advocacy purposes and not as a reflective evidence base per se), we offer a critical analysis thereof, to not only address a lacuna in the literature but also to raise questions about claims that have been largely left unchecked.

## Methods

The existing literature has explored the key characteristics of various innovative financing mechanisms, such as social bonds, AMCs and APCs, which have been used to finance responses to discrete global health challenges. However, a review of the applicability of innovative health financing instruments for PPPR and its ability to fill gaps towards the estimated US$10.5 billion annual shortfall has not previously been attempted.

To examine this relationship, we first scoped online and grey literature on innovative financing for PPPR to identify claims of its potential benefits and associated risks, drawbacks and challenges. We conducted a series of online searches from four databases – Scopus, PubMed, Google Scholar, and Google, with a focus on literature published between January 2020 and April 2024. The following combinations of search terms were used: innovative financing mechanisms/tools/instruments/models for pandemic preparedness/global health, innovative health financing for pandemic preparedness, pandemic preparedness financing/funding, pandemic contingency financing/funding, pandemic prevention funding/financing, pandemic preparedness bonds/loans/insurance/financing grant, and PPPR variants of the aforementioned. Search results were screened via title and abstract for relevance, based on whether the publications engage explicitly with the use of innovative financing instruments for PPPR (in general or with regards to a specific mechanism). The aim of this initial review was to provide insight into how innovative financing is being discussed in the context of PPPR and assess the landscape with a focus on concrete mechanisms used to mobilise funds for epidemics and pandemics in the past. Although there are several innovative financing mechanisms from which lessons can be learned, four mechanisms stood out via the searches regarding their relevance for PPPR.

Based on this initial review, we conducted additional searches on these four different innovative health financing instruments – the IFFIm, the PEF, the Gavi Ebola APC, and the Gavi COVAX AMC – premised on: 1) their prominence in the literature and relevance for PPPR; and 2) the variation in operational models that they represent. To evaluate their applicability, we then conducted content analysis and organised the information into three categories that cover the following aspects of each mechanism: operational model / design; purported value, benefits, claims to effectiveness and achievements; and shortcomings, associated risks and issues arising from the application of these mechanisms.

The article comprises five sections, the first four of which cover the selected mechanisms – the IFFIm, the PEF, the Gavi Ebola APC, and the Gavi COVAX AMC, followed by a discussion (Section V). Each of the first four sections, dedicated to a discrete innovative financing mechanism, is divided into the following subsections: 1) a brief outline of the type of innovative financing mechanism reviewed (i.e., vaccine bonds, pandemic bonds, APCs and AMCs); 2) the design of the specific mechanism reviewed (e.g., the Gavi Ebola APC); 3) its purported value and achievements; and, 4) its shortcomings. For ease of reference, we have provided a summary table of the main findings of the analysis for the innovative financing mechanisms reviewed in this article (see Appendix).

## Results

Our review revealed a scarcity of quality analytical studies on the use of innovative resource mobilisation models for PPPR. The wider body of literature on innovative financing mechanisms for global health is fragmented, as publications tend to focus on discrete mechanisms, including detailed analyses on APCs [17], vaccine bonds (IFFIm) [14], the Gavi COVAX AMC [18], or the PEF [19], some of which draw comparisons between the APC and AMC models [17, 20] and discuss alternatives (e.g., Benefit-Based Advance Market Commitments – BBAMC) [20]. A recent contribution reflecting on the development of new global health financing institutions offers a critical take on the Gavi COVAX AMC and touches upon the failings of the PEF (among a wide range of funding institutions and agencies discussed), raising questions about the role of these mechanisms in the changing global health financing landscape [21]. However, none of these publications focus specifically on the suitability of innovative health financing instruments for PPPR and current PPPR budget estimates, nor do they go beyond AMC-APC comparisons to critically examine a wider range of relevant instruments, so as to better understand their historical contribution to and prospective implications for PPPR efforts more broadly.

In addition, there is rarely a recognition of the potential of these mechanisms to mobilise funding of the magnitude required to address a specific objective and the contribution we could expect towards the proposed US$10.5 billion annually in additional ODA as recommended by the World Bank and WHO [1]. As the analysis below demonstrates, the optimistic rhetoric surrounding innovative financing for PPPR conceals not only the limited contribution these mechanisms could make relative to global PPPR funding estimates, but also their ineffectiveness in deploying the resources mobilised.

### I: The International Finance Facility for Immunisation (IFFIm) – Vaccine Bonds

#### Vaccine bonds

Social bonds are described as innovative financing tools “for mobilising private capital for the public good”[22]. Alongside other debt instruments using similar financing structures (green and sustainability bonds) [23], social bonds are touted as “an important part of global fixed income markets,” as an increasing number of investors seek opportunities to “align their portfolios with their financial goals and internationally recognized sustainability goals” [24, 25]. Akin to conventional bonds, social bonds offer fixed returns for investors but proceeds are “used exclusively for social causes,” and “address socioeconomic issues that other capital market mechanisms do not” [22]. Thus, they are perceived to attract investors “looking for a socially responsible investment with a clear, unambiguous purpose and a portfolio diversification opportunity with attractive risk-adjusted returns” [22].

The IFFIm has been credited with creating “the world’s first social bond in 2006,” which became “the template for the subsequent high-growth green, social and sustainability bonds market, which also requires the use of proceeds to be disclosed” [22]. The IFFIm raises money towards vaccination efforts via ‘vaccine bonds’ [26, 27]. These specialised bonds offer a “direct link to vaccines via Gavi” [28]. For instance, in response to the Covid-19 pandemic, IFFIm vaccine bonds channelled funds to Covid-19 vaccines, via Gavi and COVAX [27].

#### The IFFIm’s design

IFFIm was launched by Gavi in 2006 to raise funds for the achievement of the Millenium Development Goals and fill an estimated funding gap of “$30 billion to $70 billion per year until 2015” [29, 30]. It is a financial ‘frontloading’ mechanism intended to increase the availability and predictability of funding for Gavi’s vaccination programmes [31]. Frontloading is the process of “shifting financial resources from the future to the present,” by which long-term donor commitments are transformed (through vaccine bond issuances) into immediately available funding for Gavi [32]. As such, it seeks “to provide a sizeable volume of development financing into an issue area in the immediate to short term, notwithstanding the fact that the intervention programmes may continue to run into the medium to long term” [31]. Vaccine bonds issued by IFFIm are backed by “long-term, irrevocable and legally binding pledges from 11 sovereign governments” (Australia, Brazil, Canada, France, Italy, The Netherlands, Norway, South Africa, Spain, Sweden and the UK) for a period of up to 29 years (until 2037) [33].

#### Purported value and achievements

The IFFIm aims to provide the following benefits:

- “(1) increased purchasing power (i.e., lower costs through large bulk vaccine purchases); (2) allowing procurement organizations (e.g. GAVI Alliance, UNICEF) to enter into long-term purchase commitments which can significantly reduce the unit cost of vaccines; (3) reductions in the long-term disease burden by front-loading of immunization, which increases ‘herd immunity’ in affected communities;^1^ and (4) improved planning and budgeting in recipient countries” [34].

The IFFIm delivers value to GAVI by increasing the volume of funding immediately available for its programs and initiatives, fast-tracking vaccine delivery, offering flexibility to use funding as and when needed to achieve short- and long-term objectives, increasing the long-term predictability of funding and helping to “drive down vaccine prices and secure supply” [32]. Additionally, donors benefit from a lower-cost way to spread their contributions while their pledges are put to work faster with immediate impact on saving lives [32, 35]. For instance, without IFFIm, if a donor pledges US$100 million paid in US$10 million tranches per annum, Gavi would only be able to spend the US$10 million paid annually and “would have to wait 10 years before seeing its full impact” [35]. While this seems compelling at face value, especially in the context of childhood immunisation, the speed of making funding available is not the sole key determinant of a mechanism’s effectiveness and suitability for PPPR, as discussed below.

In terms of PPPR, Crocker-Buque and Mounier-Jack’s analysis of IFFIm stakeholders’ perspectives on the facility reveals that “an IFFIm-like mechanism” would be suitable for pandemic preparedness as it could “urgently raise funds,” e.g. in the context of a pledging conference, to respond to an infectious disease outbreak [36]. In December 2022, the IFFIm positioned itself as “an ideal vehicle to support future pandemic preparedness financing,” announcing its collaborative work with Gavi “to design an approach to leverage the IFFIm structure for a contingent financing mechanism that would enable donors to expedite funding to Gavi in response to a future emerging health threat” [37]. This announcement follows earlier claims by Gavi that IFFIm’s frontloading approach “could improve global pandemic preparedness now, while allowing donor governments to spread the cost” [4].

**Fig. 1:**
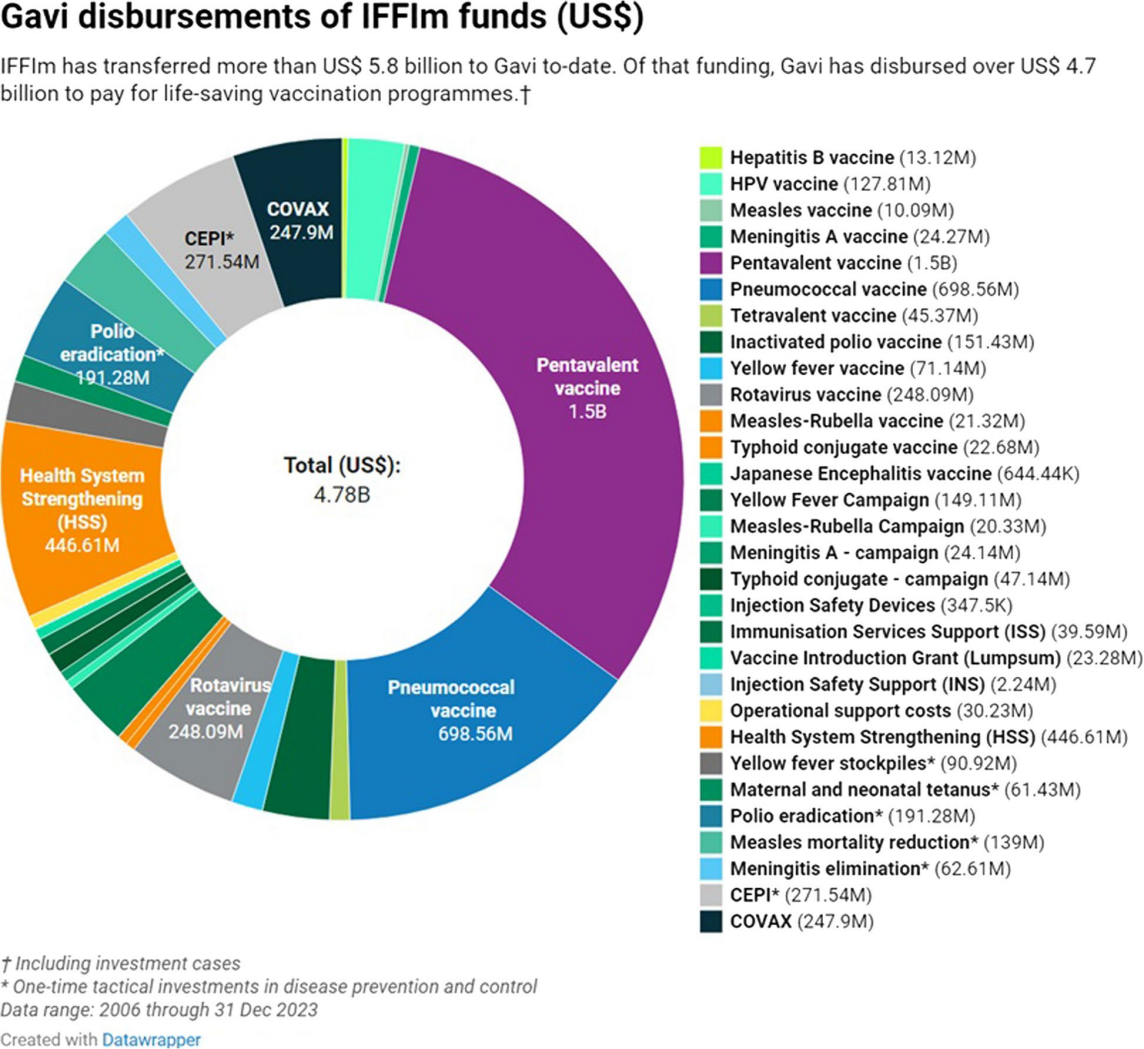
Gavi disbursement of IFFIm funds (Data range: 2006 through 31 December 2023)

The IFFIm’s ambitions are backed up by a plethora of self-reported achievements and claims to effectiveness. In the 18-year period since the IFFIm issued its first bond (as of June 2024), it has raised approximately US$9.7 billion through the long-term pledges of 11 sovereign donors [38]. In so doing, it claims to have provided “immediate funding for initiatives today that will avoid the need for much larger health-related expenditures in the future, while still spreading its payments over that longer horizon” [39]. Initially supporting Gavi’s childhood vaccine programmes, the IFFIm has expanded its mandate to mobilise funding for Ebola response, CEPI (the Coalition for Epidemic Preparedness Innovations), COVAX and PPPR [4, 37, 39, 40]. Up until December 2023, IFFIm’s contribution to Gavi’s vaccination programmes was US$5.8 billion, comprising 18% of Gavi’s overall funding from 2006 to 2023 [41]. Figure 1 (from IFFIm’s website) illustrates details of Gavi’s disbursement of IFFIm funds [42].

The IFFIm prides itself on making aid-financing history by initially securing up to 23-year legally binding commitments from donors [43]. IFFIm self-assessments claim that in the period 2006–2024, it has provided over “one-sixth of Gavi’s programme funding,” helping it to vaccinate over “1 billion children, saving 17 million lives and reducing child mortality by half across 73 low-income countries” [26, 35]. These claims of effectiveness have received support from Gavi, suggesting it “saves more lives faster,” [44] and the WEF, suggesting IFFIm helped “protect an additional 99 million children sooner from vaccine-preventable disease” [4]. Gavi highlights that vaccine bond proceeds “help ensure predictable funding and more efficient operations” and that IFFIm funds enabled it to encourage “country demand for the five-in-one pentavalent vaccine, enlarging the size of the market, attracting new manufacturers and reducing prices” [44]. The latter is one of 13 vaccine introductions the IFFIm has supported [45].

In addition, the IFFIm draws attention to its funding being used to “support[t] health system strengthening in lower-income countries and investment cases such as the COVAX AMC” [41]. In 2020, Gavi’s replenishment took place in the midst of the Covid-19 pandemic, rendering the IFFIm well-positioned to take the role of a mechanism to resolve the COVAX AMC financing gap [14]. The IFFIm became a “vehicle through which donors can support the Gavi COVAX AMC,” [41] to make Covid-19 vaccine doses available to populations in “lower-income countries that would otherwise struggle to access them” [4]. When the first social Covid-19 bonds were issued, a former IFFIm chief expressed a belief in their potential to “speed up the pace of economic recovery for sectors affected by the pandemic by maintaining basic services and increasing capacity and efficiency in the provision of healthcare services and equipment and medical and vaccine research” [22]. Generating US$975 million to this end and disbursing over US$247.9 million to the Gavi COVAX AMC up to 31 December 2023 (see Fig. 1 above), the IFFIm and Gavi claim its vaccine bonds allowed donors to fast-track funding and facilitate ‘equitable’ global access [41, 44]. In October 2024, the “IFFIm priced a new US$ 1 billion three year bond to help protect millions of children against preventable disease and prevent the next pandemic” [40, 46]. At best this could help build capacities to respond to epidemics and natural zoonosis spillovers (emerging infectious diseases) before they become pandemics, but this might be better labelled as preparedness rather than prevention since these funds would operate more like a surge fund or response capacity than a preventative measure, which would require a greater focus on upstream determinants.

#### Shortcomings: Behind the façade of IFFIm’s proclaimed success

Proponents of the innovative financing agenda for international development often cite the IFFIm as an “emblematic success story” [14]. However, as a 2023 Society for International Development (SID) report remarks, the IFFIm “has not been subjected to much scrutiny across the years” [47]. Remarkably and unlike its inefficient health-related bond counterparts, the IFFIm “has continued to operate according to its original design” despite the economic shockwaves induced by the 2008 financial crisis and the Covid-19 pandemic [47]. The IFFIm had remained immune to scrutiny until a 2022 study published by Hughes-McLure and Mawdsley. Adopting a *follow the money* methodology,^2^ Hughes-McLure and Mawdsley (2022) “set out a significant, evidence-based challenge” to dominant claims about IFFIm and innovative development finance more broadly [14].

##### Not as effective (as claimed)

As part of their analysis they critically examine whether the IFFIm delivers on the following claims: 1) “to front-load long-term donor commitments,” fast-tracking funding made available to Gavi in comparison with “more traditional rounds of donor pledging;” 2) its low funding costs by virtue of its AAA credit rating, allowing for “low spreads on its bonds;” and, 3) its ability to “leverage or mobilise private-sector funds” [14]. The authors find that IFFIm delivers on its first claim to make funds available to Gavi sooner than might otherwise happen. Hughes-McLure and Mawdsley’s “research confirms this significant front-loading effect, especially in IFFIm’s early years where Gavi receipts from IFFIm far exceed government grants” [14]. According to the authors, “IFFIm’s front-loading effect has been substantial, with immediate benefits to any children that Gavi would not otherwise have been able to vaccinate [with] [d]onor decisions to fund IFFIm hav[ing] contributed to saving an estimated 2.9 million of the 14 million lives saved by Gavi” [14]. While Hughes-McLure and Mawdsley demonstrate that the IFFIm’s frontloading model has made fast-tracking funds for Gavi’s vaccination programs possible, they uncritically take IFFIm’s claims of lives saved at face value, irrespective of the fact that other health measures impacted on outcomes. In the absence of independent evaluation of these claims, it is difficult to ascertain the impact of frontloading in terms of lives saved, leaving the IFFIm’s claim to saving more lives through fast-tracking funding poorly substantiated.

IFFIm’s second key claim to “low funding costs” is challenged by Hughes-McLure and Mawdsley’s *follow the money* analysis, which reveals an “expensive model with a plethora of mechanisms through which money is transferred to private financial actors” [14]. While “[p]ayments on bonds and fees represent a significant profit opportunity with low risk for bondholders, financial institutions, and professional services firms,” these favourable conditions are possible “because public finance protects investors” [14]. Conservative risk management and policies aimed at extenuating risk for investors, detailed by the authors, “limit the amount of front-loading of funds to Gavi while providing a high level of protection for investors,” effectively using government aid “to mitigate risk for private capital” [14].

IFFIm’s third claim to mobilising private-sector funds is dismissed by Hughes-McLure and Mawdsley. Despite leveraging private-sector funds and making these borrowed funds available to Gavi early, which is indeed useful to facilitate quicker response, the authors reveal “there is no aid additionality in IFFIm’s financing model” [14]. Contrary to catalysing additional aid funding, it merely channels part of Gavi’s funding, sourced from publicly financed donor government grants, to the private sector “in the form of interest costs of borrowing from capital markets and fees” [14].

##### Not as good a deal for donors and beneficiaries

In addition to shedding light on “precisely who benefits and by how much,” Hughes-McLure and Mawdsley’s geopolitical analysis of IFFIm’s origins demonstrates that it operates within a small and close-knit network of political and financial actors [14]. By “rely[ing] on technocratic financial management [which weakens political control over and limits accountability] and demand[ing] considerable technical complexity,” innovative financing instruments, such as the IFFIm, “close down and limit future policy options” [14]. Relatedly, SID’s 2023 report highlights that “bond issuance operations are conducted by financial institutions that are predominantly based in the United Kingdom or in the Global North while state actors and technical advice from the countries that are supposed to be IFFIm’s beneficiaries are not present” [47]. This means that “the financialisaton of global health and development is shaping economic geographies [in] which material rewards are distributed unevenly, concentrating in a (small number of) financial centres in the Global North” [14, 47]. Moreover, concentrating the financialisaton of aid by the North in the North creates considerable room for conflicts of interest. As SID concludes, Hughes-McLure and Mawdsley’s critical financial analysis of the (geo)economic consequences and implications of IFFIm’s financial model revealed “evidence of nontrivial private profit making, hiding in plain sight, at the expense of beneficiaries and donors” [47].

##### Further problems with the IFFIm

On the surface, the IFFIm appears to be a revolutionary mechanism setting a gold standard of innovative financing. According to the WEF, in the present “economic climate, this kind of frontloading could improve global pandemic preparedness now, while allowing donor governments to spread the cost” [4]. However, this is not weighed against strong evidence that costs will be saved in the future, since long-term returns on investment for PPPR are notoriously difficult to calculate and often oversimplify returns [48].

The IFFIm has been relatively successful in terms of resource mobilisation compared to other innovative tools discussed below. However, US$9.7 billion over 18 years still pales in comparison to the estimated US$10.5 billion *per annum* being asked for PPPR globally [1]. Furthermore, Hughes-McLure and Mawdsley observe that IFFIm disbursements in 2020 and the first half of 2021 conformed to a pattern “of raising considerable sums of government aid (almost $2 billion), matched by substantial bond issuances ($1.5 billion) to front-load public funding and refinance debt, and a significantly smaller sum reaching Gavi” [14]. This highlights the importance of setting reasonable expectations of the relative contribution that innovative financing could make towards PPPR budgeting.

The IFFIm also reveals specific concerns common to public–private partnerships, namely: lack of accountability, the potential for excessive private sector profiteering at the expense of public donors and beneficiaries, and control concentrated in the Global North. As detailed in the Discussion, the use of bonds, with which governments underwrite all risk to incentivize pharmaceutical companies to produce vaccines, is a massive liability for PPPR, requiring the public to shoulder all risk whilst profits accrue to Pharma. Disconcertingly, as Hughes-McLure and Maudsley’s observe:

“Despite the financial and political costs of IFFIm’s financialized innovative model, donors continue to consider it an effective and valuable model for funding vaccines at a global level, while investors, financial intermediaries, and other professional services firms continue to benefit from a good low-risk source of financial rewards” [14].

Finally, the IFFIm continues a concerning precedent, in which claims to effectiveness are left unchecked and reproduced uncritically in the grey and academic literature to the point of reaching a taken-for-granted status. This is perfectly illustrated in Hughes-McLure and Mawdsley’s critique of the IFFIm. While their *follow the money* analysis undermines other central claims to IFFIm’s effectiveness, they concede without question that the IFFIm has lived up to its claim of saving more lives (and as many lives as claimed) through fast-tracking health financing – claims which are entirely premised on information published by the IFFIm, Gavi and their affiliates. Insufficient independent scrutiny has allowed the IFFIm stakeholders to create a circular evidence and citation base with self-referential claims to effectiveness. Thus, the IFFIm success story has taken on a life of its own by virtue of its uncritical reproduction in the literature on innovative financing. As will be illustrated below, this is a recurring issue that conceals the trade-offs and opportunity costs of other potential innovative financing mechanisms for PPPR, carrying the risk of redirecting scarce funding from global and national health priorities of greater burden. This is a broader concern that has emerged in a separate analysis of the estimated cost and financing requirements associated with the PPPR agenda [48].

### II: The Pandemic Emergency Financing Facility (PEF) – Pandemic Bonds

#### Pandemic bonds

Pandemic bonds are specialised bonds “where the capital raised is earmarked for responding to pandemic outbreaks” [49]. Like most regular bonds, they function as a loan between investor and issuer, where the former makes a capital investment for a specific period of time, during which they receive coupons (periodic interest payments) from the latter [49]. What distinguishes pandemic bonds from regular bonds is that the repayment of the initial investment at maturity depends on whether a pandemic occurs. Namely, in the event of a pandemic before the maturity date, investors would lose all or part of their capital, which is used to finance the outbreak response. Yet, if no outbreak occurs their investment would be repaid on the maturity date [49]. The first pandemic bonds “transferring pandemic risk in developing countries to the financial markets” were introduced by the World Bank in 2017, almost a century after the last major pandemic (Spanish Flu), with the aim to raise funds from private investors to financially support the Pandemic Emergency Facility – an innovative financing mechanism launched in 2016 [49].

#### The PEF’s design

The 2014 Ebola outbreak in West Africa highlighted not only the lack of capacity among some LMICs “to deal with such a severe disease outbreak” but also the limited ability of the international community to rapidly mobilise funding [50, 51]. In response, the World Bank developed “an innovative, insurance-based financing mechanism” called the Pandemic Emergency Financing Facility (PEF), in consultation with WHO and public and private partners “to provide surge financing for response efforts to [the world’s poorest] countries affected by a large-scale outbreak to prevent the outbreak from reaching pandemic proportions” [52]. The financial structure of the PEF comprised two complementary windows – a cash window and an insurance window, through which the financing could be provided [52].

The cash window provides a discrete source of funding to the PEF, independent of the insurance window (and associated pandemic bonds) [49]. The PEF cash window could provide fast financial support (within days of approval by its Steering Body) to eligible countries fighting disease outbreaks, which may not be covered by the insurance window [49]. The PEF insurance window only provided coverage for viruses with pandemic potential, i.e. “large-scale outbreaks of a pre-established group of diseases [on the WHO priority disease list] identified as likely to cause major pandemics [including] pandemic Influenza (new or novel influenza A virus), Coronaviruses (e.g. SARS, MERS), Filoviruses (e.g. Ebola, Marburg), Crimean Congo hemorrhagic fever, Rift Valley fever, and Lassa fever” [52]. Under its insurance window, the Facility had capacity to provide payments over a three-year period to a maximum of US$425 million for all qualifying outbreaks combined [52]. Specifically, “the World Bank sold pandemic bonds to the value of $320 m and swaps to the value of $105 m” [49]. Despite efforts to get a PEF 2.0 off the ground following the initial period (July 2017 to June 2020) [53], the PEF’s insurance window was not renewed when the bonds matured and officially closed on 30 April 2021 [51].

#### Purported value and achievements

According to the World Bank’s PEF Fact Sheet, the Facility’s *cash window* paid out US$61.4 million to fight two Ebola outbreaks in the Democratic Republic of Congo (DRC) – US$11.4 million for the 2018 outbreak, and US$50 million for the 2019 outbreak [51]. The funding was transferred from the PEF to WHO and UNICEF at the request of the DRC government [51]. Indo-Pacific Health Security – a partner of the World Bank, claimed that these payouts proved the PEF’s effectiveness, as the 2018 outbreak was effectively contained,” and a shortfall addressed in the 2019 outbreak [54]. While the PEF’s *cash window* appears to have helped fill gaps in early outbreak containment, this funding is completely separate from the *insurance window*, which is where the PEF’s main claim to innovation (and majority of funding) lies.

The PEF’s *insurance window* was triggered once, on 27 April 2020, when the Steering Body of the PEF announced that US$195.84 million were allocated to “64 of the world’s poorest countries with reported cases of COVID-19 [with] [s]pecial attention… given to areas with the most vulnerable populations, especially in fragile and conflict-affected countries” [55]. These funds were intended to provide support to frontline health workers, PPE (personal protective equipment) and medical equipment in these countries [55]. By 20 September 2020, the PEF claimed that the “insurance payout had been transferred to the beneficiary countries, providing additional financial support to their COVID-19 response” [55]. Nonetheless, the PEF track-record of payouts has been heavily criticised, contributing to its widespread depiction as a ‘failed’ innovative financing instrument in the grey and scholarly literature.

#### Shortcomings: The failure of pandemic bonds

The PEF did not live up to its stated objective “to tackle a financing challenge critical to managing severe disease outbreaks with pandemic potential” [50]. Notably, the pandemic bonds notoriously failed to deliver surge funding in the face of the 2018 and 2019 Ebola outbreaks in the DRC, and when they did for Covid-19, it was considered insufficient and late [56, 57]. The PEF’s failings have been attributed to its poor design, among other critiques of the Facility, detailed below.

##### Design flaws

The World Bank’s former chief economist, Lawrence Summers, slammed the PEF’s bad design, calling the Facility “an embarrassing mistake” and a symptom of “financial goofiness” within the institution. He blamed the design flaws on “goofy governments who wanted to have an initiative for the G-7,” “World Bank officials who didn’t understand the first thing about finance but … loved the word ‘private sector involvement’” and “bureaucrats at the bank who were looking to make their careers by having had a major innovation.”[58] Summers criticised the PEF for failing in its intended function to provide insurance coverage to LMICs against a potential disease outbreak, and failing to deliver quick payouts to international responders and affected countries rather than waiting for international donors to respond (alluding to the PEF’s insurance arm’s failure to pay out in the face of the Ebola outbreak in the DRC) [58]. Likewise, prior to the PEF’s insurance window being triggered for Covid-19, Olga Jonas – a former World Bank economist and senior fellow at Harvard Global Health Institute – stated “what’s obscene is that the World Bank set it up this way. It waits for people to die,” with bond terms “so convoluted, it is not at all clear whether they will pay out at all. It is too little, too late” [57].

As detailed above, the provision of ‘pandemic insurance’ to developing countries is premised upon “a set of predetermined criteria [being] satisfied for an outbreak to be categorized as a pandemic that would trigger a PEF payout” [59]. Zhu explains that the bond payout conditions were “poorly designed given the nature of pandemics,” as they “were too strict and slow to be triggered” [19]. Key trigger conditions included: 1) payouts are considered 12 weeks after the start of the pandemic/epidemic; 2) at least two countries affected by at least 20 deaths each; and 3) case growth rate over a two-week period – a complex calculation, requiring two weeks of additional data and analysis, which means the disbursement of funds would take a minimum of 14 weeks, not taking into account that relying on data reporting from affected countries may not happen in a timely manner for “political reasons” or late detection [19, 60].

All of this meant that “the theoretically earliest date” for PEF insurance funds to be released for Covid-19 was 9 April 2020 – over four mounts after the first confirmed case [19]. On that very date, Ritchie and Plant issued a damning critique of the PEF’s failing to disburse funds in a timely manner due to the lengthy period of time “that has to elapse before the criteria are even assessed”, even though the Covid-19 outbreak was declared on 31st December 2019 when China informed the WHO of a new outbreak “for the purpose of the bonds” [60]. Nonetheless, it took an additional two weeks for the PEF to allocate the US$195.4 million to over 60 low-income countries on 27 April 2020 to help them fight the outbreak [52].

As previously noted, the PEF had already failed to pay out twice prior to the Covid-19 pandemic. Despite the fact that these bonds were created to address the need to respond to pandemics quickly, Ritchie and Plant highlight that:

“in the 2014 Ebola outbreak, WHO estimated that the cost of responding effectively was $5 million in the first month. Three months later, this had risen to $71 million, and then to $490 million a single month after that. Roughly, in the same period of time it takes for a PEF payout to occur, the cost of tackling Ebola increased a hundredfold. Even with a big margin of error around these numbers, it is clear that the speed of payouts would not have been ideal” [60].

The failure to pay out during the 2019 Ebola outbreak has been attributed to PEF trigger conditions relating to the ‘number of recorded deaths’ from relevant diseases, which is “problematic in countries with limited ability to accurately track and record cases” [60]. The PEF stipulated “a payout of $45 million for Ebola if the officially confirmed death toll reaches 250,” provided that at least 20 deaths occur in a second country [61]. On 26 July 2019, the Financial Times reported that Ebola fatalities in the DRC had reached 1,700 [62]. However, the pandemic bonds failed to pay out during the 2019 Ebola outbreak in the DRC because the criteria, mentioned above, required 20 fatalities from the virus in a second country [60]. While there were a few recorded deaths in neighbouring Uganda, they were insufficient to meet the threshold, even though fatalities may have been underreported [60]. Further critiques shed light on the lack of disbursement planning by the PEF. Following the Ebola outbreak in 2019, when the PEF’s cash window disbursed money, the DRC “had to submit a clear response plan and specified implementing arrangements with their request for funds” [63].

##### Falling short of PPPR financing needs and unfit to finance preparedness

Madhav, Oppenheim, and Gallivan (2017) addressed USAID and the Centers for Disease Control and Prevention (CDC) concerns about scale and timing of funding, namely that “the maximum total coverage over a three-year period is US$500 million [which is] much lower than the estimated US$3.8 billion cost of the multinational response to the 2014 West Africa Ebola epidemic” by noting that since the PEF was “designed to trigger early in an outbreak, the anticipated funding is less than would be required for a full-fledged response once a widespread pandemic is under way” [64]. While this may be true (and even though innovative financing mechanisms are only meant to supplement other sources of funding), US$500 million over three years are a drop in the ocean, compared to the estimated PPPR financing demands of US$10.5 billion per annum (even without a pandemic being declared).

It is arguably not possible for the PEF to finance PPPR, because it is a surge fund and “not a substitute for investments on preparedness,” as the World Bank stated in 2016 [65]. This relates to yet another weakness in the PEF’s design examined by Zhu, who describes the fund as “medically ineffective” as it wrongly targeted only pandemic-hit World Bank International Development Association (IDA) countries as potentially eligible funding recipients [19]. Zhu argues that while “massive resources should be devoted to the epicenter to try to prevent it from evolving into a pandemic,” PEF funding “should go to all IDA countries when the perceived risk of pandemic is high, before there are domestic positive cases” [19]. Once a pandemic hits IDA countries, effective yet relatively inexpensive, “measures to minimize viruses entering IDA countries, by increasing IDA countries’ resilience, maximizing their short-term healthcare capacities (in prevention, diagnosis and treatment)” would “lose their maximum effectiveness” [19].

The type of surge funding provided by the PEF therefore concerns only the ‘R’ (response) in PPPR, making it unsuitable for holistic public health approaches. Ignoring ‘prevention’ and ‘preparedness’ is particularly problematic in light of Madhav, Oppenheim, and Gallivan’s (2017) observation that the utility of risk transfer mechanisms such as the PEF, whose insurance window offers a financial injection “to help insured parties rapidly scale up disease response activities… depends, in large part, on the absorptive capacity of the insured party” [64]. That is to say, the success of PEF-like risk transfer mechanisms is premised on LMICs having “the ability to use insurance payouts effectively to access additional human resources (clinicians, community health workers), personal protective equipment and other medical equipment consumables, and vaccines and therapeutics, from either domestic or international resources” [64]. This was also recognised by the World Bank. In an effort to address the PEF’s lack of focus on preparedness, the World Bank planned to “further encourage IDA countries and other partners to prepare or update their outbreak response plans, which are an essential element of preparedness” as part of the PEF’s implementation, further noting that “[c]ountries need resilient health systems with quality universal primary care and strong public health capabilities, with regional networks that can take disease surveillance and detection to scale” [65]. Given that the 2014 Ebola outbreak revealed that LMICs’ limited absorptive capacity is a critical prohibitive factor in effectively responding to severe outbreaks, an innovative financing pandemic facility that focuses solely on response and relies on early activation (which did not happen in the PEF’s case) is a poor investment of PPPR and global health funds.

##### Financial inefficiency and private profiteering at the cost of development and health

Although the World Bank did not advertise the exact terms of the pandemic bonds, former World Bank economist Olga Jonas’ endeavour to “ploug[h] through the confusing 386-page bond prospectus” revealed that “the PEF has cost much more than it has brought in” [61]. As Jonas details in her critique of the PEF’s failure to payout for Ebola:

“The PEF has already paid around $75.5 million to bondholders as premiums, but has not disclosed how much they have been paid in interest — and it is set to pay much more. However, outbreak responders have received just $31 million from the PEF, and the much-touted potential payout of $425 million is highly unlikely. Twice as many investors signed up to buy pandemic bonds as were available” [61].

According to Jonas, at least in the case of Ebola, this made the PEF “a good deal for investors, not for global health” [61].

Zhu also takes issue with the financial inefficiency of the PEF, noting that “the $320 million face value of the bond could never be fully utilized [as] additional conditions are imposed to cap the percentage of fund used for pandemics with different perceived damage indicated by fatality” [19]. Due to “the loss cap of 16.7% for the Class A bond….the de facto maximum assistance available is only $195.8 million rather than $320 million during a full-blown pandemic like Covid-19” [19]. In sum, “the high cost and low usage contribute to inefficient allocation of resources,”[19] signalling a high opportunity cost.

As Global Health Advocates’ detailed examination of the PEF suggests, “in order for the PEF model to work, it has to be attractive to investors, which means it is designed to reduce the likelihood of pay-outs, one of the risks being that they come in too late in the outbreak cycle and end up making a smaller difference than if the resources had been unlocked earlier” [66]. Estimates that investors “could have received up to US $64 mn in profits funded by public money” up to July 2019 illustrate the lucrative deal the PEF offered for bondholders [66]. In addition, the PEF insurance window “incurs a US $19 mn loss per year – the difference between the payment of insurance premiums and other fees (US $39 mn per year) and the expected coverage of the insurance (US $20 mn per year)” [66]. The cost of this yearly loss is ultimately born by the intended IDA beneficiaries of the PEF, the amount of money from IDA and donor countries (Germany and Japan) “used to finance coupons would have otherwise gone directly to reducing poverty and reaching the SDGs in IDA countries” [19, 66]. This has led Global Health Advocates to conclude that “Under the narrative that public money should be used to catalyse additional investments from private finance, the PEF shows how the private sector has succeeded in mobilising public funds to increase its sales of financial services, in this case in the shape of insurances” [66].

A related concern with the financial efficiency of the PEF raised by Zhu is its unsustainability, given that the fund for premium and interest was sponsored by donations from two countries (Germany and Japan), which could not be expected to do so permanently despite initial plans for annual replenishment of the *cash window* that never came to fruition [19]. This is yet another serious omission in the PEF’s design, which highlights the problematic role of replenishment models in PPPR financing.

##### The PEF’s closure

On 8 April 2019, the World Bank announced it was “seeking to hire a modelling agent to work on the development of the next phase of the Pandemic Emergency Financing Facility (PEF 2.0)” [53]. Suggestions for the PEF 2.0 included increasing its financial sustainability and flexibility in its disbursement threshold. The World Bank “encourage[d] as many countries as possible to become contributors to the PEF cash window and insurance window so that the PEF 2.0 is financially sustainable” [67]. Yet, on 6 June 2020, the Financial Times reported that the “World Bank has shelved plans for a second sale of pandemic bonds after the first drew criticism for being too slow to pay out aid to poor nations suffering from the coronavirus outbreak” [68]. Subsequently, on 30 April 2021, the World Bank announced the official closure of the PEF [69]. The World Bank did not provide an explanation but its failure to trigger timely payouts for Ebola and Covid-19 seem to be the most plausible explanations, given the concerns raised by former Bank officials. Lawrence Summers noted that the PEF “ought to be studied, and there ought to be careful and rigorous reflection when people call for innovative finance involving the private sector,” further suggesting that the World Bank needs to learn to tell the difference between poorly designed schemes and useful innovative financing mechanisms [58].

The Centre for Disaster Protection singles out four lessons from the PEF’s failure: 1) “Getting money ‘in’ is only half the issue—don’t forget to plan how to get money ‘out’ to the people who need it most, when they need it”; 2) “Make sure that you will reach the poorest and most vulnerable people as they need the money most”; 3) learn from mistakes; 4) “involve the people who matter – work with governments and communities to manage risks” [63].

In his analysis of the PEF, Erikson argues that innovative financing has already transformed humanitarian responses. However, he notes the public health sector can influence the innovative financing agenda (even if unable to supress it) by pushing for 1) publicly negotiated and open access bond triggers; 2) “disbursement criteria that place the needs of the sick before the demands of investors;” 3) consultation with communities affected by pandemics; and, 4) “engag[ing] with and interrogat[ing] the language and priorities of finance” [70]. Influencing innovative finance would require a shift from “incidental” public health sector involvement to viewing this as “first-order health prevention” [70].

### III. The Gavi Ebola Advance Purchase Commitment (APC)

#### Advance Purchase Commitments (APCs)

APCs, also known as Advance Purchase Agreements (APAs) or Advance Price or Purchase Commitments (APPCs), constitute “binding commitments to individual suppliers to purchase [as-yet-unavailable] products, if certain conditions are met” and irrespective of eventual demand [17]. APCs have become characteristic of the global response to epidemics and pandemics (e.g., Avian flu, Zika virus, Ebola virus and Covid-19), because they help mitigate the unusually high degree of demand uncertainty created by these events which discourage manufacturers from investing in product development or expanded capacity, while “hedging against R&D and manufacturing risk” and securing the availability of in demand products for buyers [17]. APCs mitigate “demand risk to suppliers by (i) guaranteeing the price to be paid, (ii) guaranteeing the volume to be purchased, or (iii) a combination of the two – amounting to a guarantee of revenue” [17]. If the product is under development, the contracts are usually contingent upon certain conditions being fulfilled, such as achieving a licence, meeting concrete technical criteria, reaching a development milestone or a trigger event, e.g. the WHO declaring a Public Health Emergency of International Concern (PHEIC) [17].

#### The Gavi Ebola APC’s design

The 2014–2016 Ebola virus outbreak constituted the “largest [Ebola] disease outbreak to date” and the third PHEIC in history [71]. Unlike six previous outbreaks since the discovery of the virus in 1976, it quickly spread from Guinea to neighbouring countries Sierra Leone and Liberia. There were 28,600 people infected and 11,325 deaths [72]. In order “to prevent a pandemic” various public and private sector stakeholders joined “efforts and resources to develop” a vaccine to combat the virus as soon as possible [71]. In late 2014, the Gavi Board approved a funding envelope – a budget of up to $390 million – “for accelerated access to Ebola vaccines including eventual procurement of licensed vaccines, vaccine delivery and support for recovery in affected countries” [73, 74]. However, the relative rarity and unpredictability of Ebola outbreaks meant that there is a poor natural market for a vaccine [75]. In response, on 20 January 2016 at the World Economic Forum in Davos, Gavi and vaccine manufacturer Merck Sharp & Dohme Corp. (a subsidiary of Merck & Co., Inc., MSD) signed a first-of-its-kind agreement, through an APC, to support the provision of a vaccine [76, 77]. Under the APC, Gavi pre-paid US$5 million to support “the development of Merck’s rVSV∆G-ZEBOV-GP live attenuated Ebola Zaire vaccine, on the understanding that it will be submitted for licensure by the end of 2017” [77]. This meant that upon approval “Gavi would be able to begin purchasing the vaccine to create a stockpile for future outbreaks” [77]. It was further agreed that while vaccine development continued, from May 2016 Merck would make available 300,000 doses of an investigational version “for use in expanded use clinical trials and/or for emergency use as needed” [77].

#### Purported value and achievements

Phase 1 trials commenced in October 2014 [71], and in October 2019 Merck’s Ervebo (rVSV∆G-ZEBOV-GP) became the first Ebola vaccine to be granted a conditional marketing authorization by the European Medicines Agency [78], confirmed by the European Commission in November 2019 [79]. In the same month, Ervebo became the first Ebola vaccine to be prequalified by the WHO – “a critical step that will help speed up its licensing, access and roll-out in countries most at risk of Ebola outbreaks” – making it “the fastest vaccine prequalification process ever conducted by WHO” [80]. In December 2019, Merck’s vaccine was announced as the first FDA-approved Ebola vaccine and “a critical milestone in public health preparedness and response” [81].

In the aftermath of the 2018–2020 DRC outbreak, Gavi claimed its APC provided “strong incentives for manufacturers” to speed up vaccine development and “ensured that doses of investigational vaccine could be deployed on a ‘compassionate use’ basis during outbreaks before the vaccine was licensed” [82]. According to the WHO, 350,000 people were vaccinated on a compassionate use basis in Guinea and in the 2018–2020 outbreaks in the Democratic Republic of Congo (DRC) [75]. Gavi chief Dr Seth Berkley described the APC as “a true success story for the Vaccine Alliance that illustrates the strength of our public–private partnership,” claiming the “accelerated development of the Ebola vaccine was possible thanks to a first-of-its-kind agreement between Gavi and the vaccine manufacturer, which set a precedent for fast-tracking development and production of vaccines against COVID-19” [82].

Nevertheless, these overwhelmingly positive self-appraisals fail to consider issues raised with regards to the Gavi-Merck agreement, the rarely discussed risks of APCs, and the subsequent issues that arose from their extensive use during the Covid-19 pandemic.

#### Shortcomings: The APC small print – failings, downsides, hidden costs and associated risks

##### Lack of transparency and potential for excessive private sector profiteering

Shortly after the announcement of what appeared to be a promising innovative financing initiative, first reactions highlighted potential concerns. Although Medicines Sans Frontiers (MSF) were “encouraged by the news” of the APC agreement, they quickly raised concerns about “how Gavi and Merck will set the price, especially in the long-term, [including a] confirmation that the final price will be set close to the cost of production” and Merck’s lack of transparency regarding the “funding contributions, R&D funding, development incentives and pricing structure”, noting that “public and philanthropic funders should [not] pay twice for the R&D for this vaccine” [83]. Similarly, Herder, Graham and Gold (2020) highlight that even though “the funding from Gavi reportedly came with an obligation to create stockpile doses of rVSV-ZEBOV and ensure the vaccine was priced affordably in developing countries,… the precise terms of the Gavi-Merck agreement [were] not publicly available” [84].

Following Ervebo’s approval, Gavi opened a new funding window through to 2025 with an estimated investment of US$178-million for a new Ebola vaccine program, as part of which the Vaccine Alliance would fund a global Ebola vaccine stockpile to be maintained at 500, 000 doses [85, 86]. Following a procurement process conducted by UNICEF (Gavi’s partner and purchasing agency) with Merck [82, 87], the price per dose determined as part of a multi-year supply agreement with the vaccine manufacturer from 2020 to 2025 was an exorbitant US$98.60 [88]. Given the “vast amount of public money that was invested in the development of the vaccine,” Merck’s share of the total development costs would have been relatively small [89]. They had used a prototype already developed by Canadian scientists funded by the Canadian government [84, 90]. In addition to the US$5 million Merck received from Gavi through the APC, they benefited from a US$176 million investment for the development of the vaccine from the US government alone [91]. Hence, it is questionable that US$98.60 per dose is an example of price fairness or the best use of vaccine funds on Gavi’s part. As MSF Access staff note, “Gavi at least should take a much closer look at the finances of this arrangement that they will have to pay for, to learn lessons for the future” [89]. The questionable details of the above agreement signal that the concerns voiced at the time the APC was announced were well-founded.

##### Delays in vaccine development, licencing and procurement

While Wolf et al. (2020) note that 5 years (from the start of the first clinical trial in 2014) “was much faster than the typical 10–15 year timeline for vaccine development and approval,” [71] the APC’s success in fast-tracking development has been heavily criticised. Ultimately, although Merck did not hold their end of the APC agreement, there were no consequences. Herder, Graham and Gold (2020) are critical of the delays in submission for FDA approval, noting that Merck missed the 2017 vaccine submission target, subsequently initiating a ‘rolling submission’ in November 2018, “when over 42,000 frontline health workers and Ebola contacts had…[already] been vaccinated” [84]. The vaccine manufacturer did not submit its product for marketing approval until March 2019 in Europe and September 2019 in the US [91]. Despite this, “Gavi was not in a position to take any punitive action since the pre-payment to Merck had already been made” [91]. Moreover, MSF Access noted that despite a US$5 million prepayment, Gavi had not yet received any approved Ebola vaccine from Merck by June 2020 [91]. As Billigton et al. (2020) claim, “in the event of a widespread epidemic or pandemic, a delay in the availability of a vaccine can result in substantial human and economic loss” and little “impact on the epidemic curve,” as evidenced by past outbreaks where vaccines arrived after epidemics had started to wane (e.g., the 2009 H1N1 influenza, 2014–2016 Ebola, and 2015–2016 Zika outbreaks) [92]. Similarly, by the time the vaccine received WHO prequalification, “there ha[d] been a steady decline in new cases of Ebola in DRC” over the previous 3 months [93]. Based on an in-depth examination of the development of Ervebo, Herder, Graham and Gold (2020) conclude that handing over the development of a promising candidate vaccine from publicly-funded researchers to Merck on the assumption that they were in a better position to bring it to market had demonstrated that the “private sector was unequal to the task” – “it was not only unnecessary to its development, but also likely slowed it down” [84].

The justification of large investments into rapid vaccine development is also called into question by its inevitable opportunity costs on other interventions. This is highlighted by the fact that all previous Ebola outbreaks resolved through non-vaccine interventions and normal epidemiological processes. As a result of limited and rapidly contained outbreaks since 2021, of the 145,690 doses that have been shipped from the Ervebo vaccine stockpile between 2021 and 2023, only 6,570 (5%) were used for outbreak response [94]. The remaining doses were repurposed for preventive vaccination to maximise cost-efficiency, but questions about the latter and associated opportunity costs remain.

##### The risk of overbuying and overpaying

The WHO highlighted a notable decline in reported new cases in affected DRC provinces from 127 to 73 new cases in the week of 22 May 2019, while “the risk of national and regional spread remained high” [95]. In the midst of this uncertainty, in June 2019 then Director of the U.S. Centers for Disease Control and Prevention, Robert Redfield Herder, raised concerns “that responders [in the DRC] may run out of the investigational Ebola vaccine” [96]. Relatedly, in July 2019, then MSF Director of Operations, Isabelle Defourny, said “DRC’s stock of vaccines is extremely low, usually less than 1,000 doses” [97]. These field reports contradicted concurrent claims by Gavi that there was no vaccine shortage on the ground in DRC with Merck supply set to cover “an additional 1,300,000 people” over 6–18 months, and Merck suggesting that there were 245,000 1.0 mL doses available for shipment at the time [97]. Contrary to these claims, Herder, Graham and Gold (2020) argue that “Merck’s supply of clinical grade rVSV-ZEBOV ha[d] not kept pace with public health needs to address the outbreak in the Congo [due to the company being unable] to produce the vaccine at its industrial scale facility” [84]. While there may have been a case for higher vaccine demand at the height of the 2019 Ebola outbreak, the situation quickly changed as clear evidence of the epidemic waning had emerged by the time Merck’s vaccine achieved WHO prequalification.

The number of confirmed new cases had dropped to only six in North Kivu and Ituri provinces in the week leading up to vaccine prequalification (6 to 12 November) [98]. This led some commentators to observe that Merck’s “plans to make another 650 000 doses available over the next 18 months,” in addition to their existing stockpile of 190 000 doses, “might not prove necessary” [93]. This also suggests that further investment in stockpiling is not value-for-money (i.e., outbreaks resolve regardless) with high opportunity costs. Thornton et al. (2022) suggest that “demand plummeted after the APA was put in place,”[17] making the Ebola outbreak a good example of how this mechanism can fail. Specifically, “purchasers, working with information available at the time, can over-estimate demand and/or its longevity, and be left buying supplies that, in hindsight, were not needed” [17]. As Funk et al. (2020) observe, “most forecasts made during the [Ebola] epidemic were later found to have overestimated the expected number of cases.”[99]

According to Thornton et al. (2022), the risk of overbuying (buying more product than needed) and overpaying (paying more than necessary for the requisite product) “when demand does not materialise” should be understood and accepted as “intrinsic” to APCs and should not preclude their use [17]. By introducing the notion of “no regrets purchasing” the authors argue that the demand uncertainty in the context of disease outbreaks “means that buyers may have to buy product that they do not end up needing – if they, and suppliers, were sure that demand would materialize, an advance commitment to buy would not be necessary” [17]. Hence, if the buyer takes the risk of overbuying in good faith, and ends up with more product than needed, it does not mean that entering into an APC was a mistake based on the available information at the time, arguing that “in the spirit of ‘no regrets,’ in a pandemic too much supply is better than too little” [17].

However, as the authors admit, “while some risk of excess supply is unavoidable, it is of course possible to commit to buying too much or at too high a price” [17]. In such cases, excess funding dedicated to vaccine development diverts scarce financial resources from higher burden health priorities [100]. ‘No regrets purchasing’ can therefore be seen as a useful euphemism for those in decision-making positions not directly affected by the impact of those decisions. However, regrets would accrue to those who have otherwise available interventions withdrawn. Less funding for malaria or infant nutrition means higher child mortality, for example. There is also the necessary caveat that purchases were based on the best available evidence at the time and that they were made in good faith. If this is determined not to have been the case it should trigger immediate concerns of conflict of interest, questionable decision-making processes, and a lack of checks-and-balances. Therefore, the risk of overbuying and overpaying should not be minimized. A more holistic and accountable approach is essential.

##### The trade-offs of extensive APC use during the Covid-19 pandemic

As mentioned above, Gavi’s Director claimed that the Ebola APC paved the way for the mechanism’s application in response to the Covid-19 pandemic. However, as detailed below, the extensive use of APCs during the pandemic exposed a host of additional concerns that caution against excessively pre-empting markets with APCs in the future. As Thornton et al. (2022) clarify, in the absence of a guarantee that a specific product will come to market, buyers who wish to secure a product in high demand recourse to using “multiple APAs for a portfolio of products” [17]. However, as the authors warn, “the extensive use of APAs by HICs [high-income countries] contributes to inequity in access by allowing these countries with greatest financial and technical capabilities to monopolize supply, at least in early stages,” noting that in the case of Covid-19, attempts to mitigate this negative effect of placing multiple bilateral APCs with suppliers through donations were made “only after they [HICs] had met their own domestic needs and did little to reduce inequity in the timing of access” [17]. In other words, there is a risk that APCs merely obscure or even exacerbate existing inequities versus resolving them – a risk that materialised in the context of the Covid-19 pandemic.

Irrespective of the impact of this on Covid-19 [100], it raises an issue for future scenarios where vaccines may have a major impact [17]. The extent of the problem is evidenced by the fact that even though R&D risk had largely passed by June 2021, and “that the 16 billion doses on order were already far above likely demand,” “another 43 APAs for another 4 billion doses” were signed [17]. Thornton et al. (2022) put this into perspective by noting that if a smaller proportion of vaccine candidates had come to market, LMICs “might still be waiting for vaccines” [17]. At the same time, contracting for larger volumes than needed and signing agreements with different suppliers in a bid to guarantee volumes via APCs is costly to buyers [20]. For instance, Canada reportedly purchased 5 times the required amount to vaccinate its entire population [20]. Consequently, the extensive use of APCs in the skirmish of HICs for potentially scarce health resources, as illustrated by the Covid-19 pandemic, once again raises the question of how prudently limited PPPR funding would be allocated through the vehicle of innovative financing mechanisms.

##### The catch in the Gavi Ebola APC and recent APC experience

The WEF cites the Gavi Ebola APC as an example of how innovative financing mechanisms can help bring epidemics to an end, by “unlocking funds that would accelerate the development of [vaccines and] other medical and health innovations” and “making fast and efficient use of funds to make health interventions available rapidly” [4]. However, questions of effectiveness aside, the findings demonstrate that the Ebola APC failed to deliver the vaccine as quickly as anticipated (and agreed) and was too late to greatly influence the epidemic (as it has been suggested that infections and demand were already going down in the DRC) [17, 98].

Much of the lauded potential for this financing mechanism is coming from two organisations influenced by manufacturers who stand to profit – Gavi and the WEF. These claims are not sufficiently backed by external evaluation or based on systematic evidence, and often resemble opinion, assumption or speculation.

In addition to being hampered by delays, Gavi’s US$5 million APC was “dwarfed” by the other investments – “the US government alone invested US$176 million to help develop Merck’s vaccine, including US$23 million to Merck to boost production” [91]. The amount mobilised through the Ebola APC is only a modicum of the cumulative financing funnelled into its development and a drop in the sea compared to the US$10.5 billion per annum estimated to be required for PPPR globally. This again highlights the limited potential of innovative financing mechanisms to bridge significant funding gaps for costly endeavours.

In addition, sweeping endorsements of the Gavi Ebola APC tend to gloss over the risks and issues associated with this mechanism. Among these issues, the lack of transparency, especially in regard to price setting, appears to be characteristic. As discussed below, similarly to the Gavi Ebola APC, “APC agreements for COVID-19… also lacked clarity on how price is to be determined, including how much is paid up-front and cannot be recovered if the candidate fails, and whether and how pricing accounts for governmental and philanthropic push investments” [20]. These issues raise major concerns of the potential misuse of limited PPPR funding through contracts that are excessively profitable for private sector actors including pharmaceutical companies, but amount to poor use of scarce funding.

Relatedly, the historical use of APCs in response to epidemics and pandemics points to a one-size-fits-all approach to dealing with disease outbreaks, typified by an overreliance on vaccines as the go-to PPPR strategy and outbreak response. The benefits of this strategy are shown here to be oversold, with conflict-of-interest concerns within the public–private partnerships and corporate associations backing them. Evidence of overall benefit, especially considering the potential alternate targets for financing and opportunity costs, is not convincingly presented. These issues resurfaced most recently during the Covid-19 outbreak [100] and will be examined in more detail in the context of the Gavi COVAX AMC below.

### IV: The Gavi COVAX Advance Market Commitment (AMC)

#### Advance Market Commitments (AMCs)

An AMC is a “forward-looking binding contract by buyers that guarantees a market for new products that meet a target product profile, at a pre-agreed price” [101]. Created to incentivise the development of vaccines, the AMC rose to prominence when it was first employed “to support pneumococcal vaccines that would protect against strains of the disease more commonly occurring in LMICs” [101]. In this context, AMCs have been used to encourage investment by vaccine suppliers to LMICs, whereby “donors commit to a fund from which a specified subsidy is paid per unit purchased by low-income countries until the fund is exhausted, strengthening suppliers’ incentives to invest in research, development, and capacity” [102]. Thus, AMCs address market failures for not-yet-available or optimised products in unattractive markets such as those in LMICs in a healthcare context, where suppliers are understandably unwilling to make large investments in upfront R&D expenses and manufacturing capacity scale-up [101, 103]. In line with the definition of innovative financing, AMCs are complementary to, but not a substitute for, other policies and interventions to support R&D for new vaccines [103].

To understand the innovative financing landscape surrounding the Covid-19 pandemic, it is important to differentiate between APCs and AMCs, as the two terms are often conflated and used interchangeably in the literature [20]. While both mechanisms are underpinned by legally-binding contracts to purchase a pre-agreed quantity of a qualifying product at a pre-agreed price and are used to drive R&D and supply of health products, “APCs contract an individual company/a specific product, whereas an AMC offers a global market commitment but not a commitment to any particular company or product” [20].

#### The Gavi COVAX AMC’s design

COVAX, established in April 2020 and closed as of 31 December 2023, was the vaccines pillar of the Access to COVID-19 Tools (ACT) Accelerator – the “global collaboration to accelerate development, production, and equitable access to COVID-19 tests, treatments, and vaccines” [104]. The COVAX Facility, COVAX’s procurement and global risk-sharing platform [105], was designed and administered by Gavi to purchase vaccines on behalf of participating countries through APCs with vaccine manufacturers [106]. Gavi also developed a separate innovative financing mechanism – the COVAX Advance Market Commitment, launched at its third donor pledging conference on 4 June 2020 [106], to “accelerate the manufacture of a COVID-19 vaccine on a massive scale and to distribute it according to need, rather than ability to pay” [107]. This architecture ensured that both the financing and the distribution of vaccines purchased by high- and upper middle-income (HICs and UMIC) and low- and lower middle-income (LIC and LMIC) COVAX member countries were separated, creating “two buyers’ and distribution clubs” [18]. Namely, self-financing HIC and HMIC countries grouped under the COVAX Facility made upfront payments into the Facility’s portfolio to purchase doses to protect their populations [15]. Respectively, it was envisioned that the COVAX AMC, primarily funded through ODA, as well as private sector and philanthropic contributions, would give the 92 participating LICs, LMICs and IDA-eligible countries under its umbrella (the so-called AMC92) access to Covid-19 vaccines equal to that of self-financing countries [15]. According to Gavi, keeping AMC funding separate from that of the COVAX Facility ensured that it was not “cross-subsidised by the funds of self-financing participants” [15].

The COVAX AMC’s operational model has given rise to scholarly debates as to whether it can be categorised as an AMC, despite being explicitly labelled as such. Thornton et al. note that “the ‘COVAX AMC’ is not technically an AMC, but a group of APAs” [17]. It was strategically labelled as an AMC due to donors’ prior experience with AMCs (especially, the Pneumococcal Conjugate Vaccine AMC) and already having an AMC budget line, which aided investment in the COVAX AMC [17]. Ultimately, the secrecy surrounding the inner workings of the mechanism precludes a thorough analysis of its design. Even though Kremer et al. (2022) set out to offer a theory, which can help explain which of “the contracts offered in the Covid-19 pandemic are appropriately labelled AMCs,” they ultimately concede that “constraints of space and contractual information preclude analysis of the merits of the panoply of contracts offered” [102]. For the same reasons, this article will not attempt to unpack these contracts or resolve the scholarly debate around the COVAX AMC’s design. Instead, we will adhere to common accepted language. To borrow a phrase from Kremer et al., we will discuss the COVAX AMC as one of “the variations of AMCs used to fund Covid-19” [102].

#### Purported value and achievements

The value of an AMC lies in its objective to make “effective vaccines at cost-effective and sustainable prices” available sooner [108], thus avoiding “substantial and costly delays in the development of and access to priority vaccines” for LMICs [103]. Specifically, the Gavi COVAX AMC was intended to encourage manufacturers to invest in production capacity which in turn would “increase supply availability and reduce the time it takes for licensed vaccines to become available, particularly to the poorest countries around the world” [107]. According to Halabi and Gostin (2023), “COVAX was intended to represent a bargain: high-income ‘self-financing’ governments would contribute monetarily toward the cost of a global vaccine distribution system and enter into advanced purchase agreements with COVAX to purchase a predefined number of doses for their own populations, accessing a large pool of products”[21]. In exchange for the insurance COVAX provided in the form of increasing HICs chances of securing vaccine doses in case bilateral deals did not supply them, the original expectation was that governments would also commit to “financial and non-financial obligations, such as supporting the delivery of vaccines in AMC eligible countries, fast-track licensure of vaccines, reporting epidemiological and virological data, and maintaining transparency about all bilateral vaccine agreements” [21].

As a result, COVAX was able to make large-scale investments and build “the largest portfolio in the world” comprising “11 vaccine candidates across four technology platforms (of which 10 received regulatory approval)” [109]. Gavi reported raising over US$12 billion in donor funding for the AMC [110, 111], allowing the latter to deliver close to 2 billion doses of the Covid-19 vaccine “and safe injection devices to 146 economies” [112]. In doing so, according to the 2023 Gavi Annual Report, COVAX “is estimated to have averted over 2.7 million deaths” in AMC countries [112]. In addition, the report states that “COVAX supplied 74% of low-income countries’ COVID-19 vaccine doses during the pandemic; and in total, 54 of the 92 AMC-eligible economies relied on COVAX for more than half of their COVID-19 vaccine supply” [112]. Regardless of notable delays and inequity in vaccine rollout, according to the Center for Global Development (CGD) and COVAX proponents, Covid-19 vaccine development and diffusion were the fastest in history and unprecedented in scale, signalling “good news” for the future of PPPR [113, 114]. Despite these claims to success and ostensible achievements, the COVAX AMC missed its target in many ways. As Gavin Yamey puts it: “It was a beautiful idea, born out of solidarity…Unfortunately, it didn’t happen” [115].

#### Shortcomings: The unfulfilled promise of the COVAX AMC

Recognising the “clear need for the world to be better prepared” for the next pandemic, Gavi outlines the key learnings COVAX can offer for future pandemic preparedness and response, with a focus on meeting foreseeable challenges such as ensuring equitable access and reducing vaccine nationalism [116]. According to Gavi, the challenge of “hoarding, export restrictions and nationalism” should be anticipated in the context of pandemic response as states would seek to “protect their own citizens first” amid “great uncertainty about which medical interventions will become available” during a global crisis [116]. In response, “COVAX’s solution was to pool demand, not just for lower-income economies, but also from wealthier nations that had resources but still lacked the power to secure bilateral deals in a supply-constrained environment” [116]. Nonetheless, the COVAX AMC fell short of meeting the challenge to ensure equitable access, posed by vaccine nationalism, while suffering from serious transparency and accountability issues.

##### Falling short of burden sharing and health equity targets

According to Halabi and Gostin (2023), the COVAX AMC’s “elaborate architecture aimed at burden-sharing and health equity fell far short of its target” [21]. The record speed at which vaccines were developed and authorized for emergency use “made little difference to LMICs [who] were effectively left behind,”[117] as vaccination campaigns in LMICs often only began after large shares of HIC populations had already been vaccinated [21]. The CGD suggests that vaccine supply shortages during 2021 and the inequitable distribution of vaccines “between countries at different levels of income, likely… cost of hundreds of thousands of lives” [114]. Even though in May 2022 the WHO reported that supply exceeded demand, it highlighted the importance of ensuring supply availability at the right times [118]. Specifically, the WHO noted that while a large number of doses from APCs and donations became available later, “COVAX experienced delays securing doses in 2021,” [118] as it had only delivered “344 million of the 2 billion doses” it had intended to distribute by the end of the year [119]. Despite delivering 1.72 billion doses by mid-September 2022, “massive vaccine inequalities persist[ed]” [114]. The WEF (2024) attributes the “succession of delays” that held up vaccine roll-out in the early phases of COVAX to a combination of “factors including a lack of up-front cash reserves, vaccine hoarding and export bans” [120]. In a similar vein, Eccleston-Turner and Upton (2021) claim that vaccine nationalism limited COVAX’s ability to raise funds from affluent donors and meet its procurement goals for vulnerable populations [121]. The authors also note that the unwillingness of many developed countries “to rely on the COVAX Facility for procurement of COVID-19 vaccines,” instead opting for a “half-in, half-out approach to multilateral cooperation,” which involved donating directly to the COVAX AMC while pursuing bilateral APCs [121], ultimately “undermined COVAX and denied [equitable] vaccine access” to vulnerable populations in poorer countries [21]. According to Open Consultants’ evaluation of ACT-A, while COVAX’s ambition was laudable, it proved “too ambitious”, as “key informants felt it was unrealistic to assume that [HICs] would purchase their vaccines through COVAX, thereby delegating authority over R&D and allocation decisions to a new global partnership” [114]. As a result of HICs not using the self-financing arm of COVAX as originally intended, “COVAX was unable to play the market shaping role it first envisioned,” and its self-financing arm was “largely perceived as a failure” [114]. In an attempt to coax reluctant HICs to engage with COVAX, Gavi made concessions that undercut the principle of equal treatment of countries underpinning COVAX [115]. Namely, it offered self-financing countries the choice “to opt in and out of certain products” (i.e. Optional Purchase Agreements) and increased the volume of product they were allowed to purchase, which raised the ceiling for self-financing countries to 50%, in comparison to 20% for AMC-eligible countries [115]. These preferential arrangements have drawn much criticism by experts [114].

It is important to note that some caution is warranted in comparing vaccination rates and required volumes between HICs and LMICs. LMICs typically have much younger populations. While in HICs 19% of the population are 65 or older, this number is 8% for LMICs and only 3% in Sub-Saharan Africa [122]. Given that the risk of falling critically ill or dying from a Covid infection skews heavily toward the elderly, the population at high risk is accordingly much lower in LMICs. Regardless of ongoing questions about the efficacy of universal mass vaccination, and the role of context in moderating the appropriateness of mass vaccination, denied access associated with the COVAX experience does throw into question the ability of innovative financing to meet the PPPR challenges associated with vaccine nationalism and access equity highlighted by Gavi.

##### Lack of transparency and accountability

Founded on a public–private partnership model that champions voluntary government-corporation partnerships and claiming to aim for transparency, “as the best way to overcome ‘market failures’ and that embraces the current intellectual property regime as a necessary driver of innovation,” COVAX “allowed pharmaceutical companies to keep vaccine contracts and prices secret, and… defended their resistance to sharing vaccine technology” [119]. In particular, Ravelo (2020) criticizes COVAX for its failure to ensure vaccine data- and technology-sharing with LMICs [123]. Notably, in addition to failing to share knowledge and technology, “COVAX did not share the power of decision-making, as it was governed by unelected officials of GAVI, and CEPI, with influence and support from HICs and private Philanthropies” [119]. Related critiques extend to CEPI, one of the co-leads of COVAX, for “the lack of transparency in its grant agreements with COVID-19 vaccine developers” [124, 125].

According to Transparency International, pharmaceutical giants like Pfizer had been subjected to limited public scrutiny, in large part due to the concerning “lack of transparency in the contracting processes of vaccines,” suggesting that a mere 6% of contracts were made publicly available (with sections redacted) and only one (0.5%) published in full [126]. A similar analysis of APCs, commissioned by The Left in the European Parliament and published by the European Commission, also highlights that the opacity around these “heavily redacted” agreements made it difficult to ascertain “whether public funds were well and fairly spent, the prices paid for the vaccines and whether there were agreements made on equitable distribution,” ultimately preventing many countries from knowing agreed prices and negotiating a fair price [117]. In addition to this lack of transparency, several contracts omitted sanctions for delays in vaccine delivery but still indemnified companies against liability by the buyers [117], raising concerns of lack of accountability. The troubling lack of transparency, governments signing unfavourable agreements with companies, and the lack of repercussions for vaccine manufacturers who did not deliver on their promises while heavily profiting from APCs that characterised the COVAX experience is reminiscent of the issues surrounding the Gavi-Merck agreement to develop the Ervebo vaccine discussed above. While COVAX reportedly justified the secrecy around its contracts by stating that it “could be detrimental to [our] future deals”, because they “contain proprietary information,” Transparency International noted that this “generally accepted” reasoning “by buyers worldwide… does not justify the complete lack of publication of contracts,” since publishing a redacted version of the contract would ensure the secrecy of proprietary information is safeguarded [127].

The secrecy surrounding a scheme of such magnitude with regards to sharing information about its inner working and how much it would ultimately cost led civil society organisations (CSOs) to voice their dissatisfaction with the “piecemeal” and late-stage CSO involvement in its “development and decision making,” and to raise concerns, especially at a time when global aid budgets were shrinking, since the AMC was likely to lock up billions of aid dollars for many years to come [107]. Médecins Sans Frontières (MSF) had expressed similar concerns regarding Gavi’s pneumococcal vaccine fund since 2008. This first AMC, which appears to have inspired COVAX, was praised as a “huge success” by Gavi, whilst MSF criticised it for the relatively high prices demanded by pharmaceutical companies [107]. Similarly to other mechanisms reviewed in this article, the AMC model used by Gavi allows for private sector profiteering at the cost of donors and beneficiaries and comes with potentially high opportunity costs.

##### The obstacles to success of innovative financing during the Covid-19 pandemic

According to an expert invited to work on COVAX, at the start of the Covid-19 pandemic, world leaders had reportedly recognised the need to turn Covid-19 vaccines “into a global public good,” as well as to do things differently so as to avoid the “moral catastrophe that prolonged” the 2009 H1N1 pandemic, where rich countries secured early access to vaccines through bilateral deals with vaccine manufacturers [18]. It appears, however, that lessons from the past had not been learned, as the same ‘mistakes’ undermined COVAX’s vaccine supply and equity goals. By striking multiple bilateral APC deals with manufacturers, HICs crowded out LMICs and “bought their way to the front of the queue,” while vaccine manufacturers did not uphold promises to deliver vaccines to COVAX, instead prioritising deliveries to high-paying countries [117]. While the Centre for Research on Multinational Corporations argues that these companies may have had a moral responsibility “to prioritise public health over profit – as some [of them] did,” stemming from the right to health and access to medicines as part of it, “the absence of contractual conditions that would have made this obligation binding gave them free rein to chase the bottom line” and led to global vaccine access inequalities [117].

In the absence of “binding international agreements and enforcement of international cooperation by capping the bilateral deals [,] international treaties were not sufficient to prevent vaccine hoarding or to specify the rights and obligations of the countries in the context of global public goods” [119]. Thus, the COVAX experience demonstrated that a voluntary scheme would struggle to persuade HICs to surrender “their political priorities and consider the morality of global vaccine equity” [119]. COVAX’s subsequent “pivot from global vaccine procurement mechanism to dose-sharing hub,” in a bid to make up for the failings of its collective purchasing efforts to share vaccines equitably and address the problem of ‘vaccine hoarding,’ only highlighted that the initiative did “*not* share… decision-making power and the knowledge and technology to produce vaccines everywhere” [119].

While COVAX hoped to ensure supply for LMICs through its own use of APCs, “a variety of exogenous factors hampered its success (e.g., greater resources at the disposal of HICs combined with their ability to mobilize their funds and sign deals quickly; the use of trade restrictions by vaccine producing countries)” [17]. This raises three concerns that will be detailed further in the Discussion. First, the mechanism was built on a presumption that the appropriate response to SARS-CoV-2 requires vaccinating ‘all’, an assumption that is questionable from a public health perspective [128–131]. Second, that issues of transparency and accountability persist, raising concerns about conflict of interest, profiteering, and blurring public and private goods. Third, assuming mass vaccinations are needed, the mechanism was unable to fulfil its mandate of fast and equitable vaccine access to LMICs, raising questions of suitability as part of a larger PPPR equation.

## Discussion

This review of innovative financing mechanisms for PPPR reveals some success in discrete areas such as incentivising vaccine R&D, fast-tracking the availability of funding for health interventions and accelerating access to medical countermeasures, but raises considerable concern as to the overall benefits and cost-effectiveness of these mechanisms. The experience of innovative health financing mechanisms applied to PPPR throws into question the prospective success of future innovative financing instruments applied to this area. Beyond the hype surrounding these instruments, our review has uncovered a patchy history of unfulfilled promises and unsubstantiated claims of effectiveness. This is not to say that these mechanisms did not achieve success in part – developing a vaccine (Gavi Ebola APC) or mobilising funding and making it immediately available (IFFIm), these should be recognised. Yet, the review does raise questions about whether the presentation by organisations behind these mechanisms conceals their limitations. In other words, they do not always do what they say they will, as they say they will, and when they say they will. In addition, it is hard to determine whether the purported successes of previous mechanisms in addressing well-defined aspects of epidemic / pandemic events could be duplicated for wider PPPR policy, which is multifaceted, longitudinal and multisectoral.

### Unsubstantiated claims to success, efficiency and achievements, and limited independent evaluation thereof preclude evidence-based assessment of mechanisms’ impact

Surveying the innovative financing literature relevant to PPPR is a discombobulating experience. The grey literature is saturated with unsubstantiated claims by organisations and their affiliates behind these mechanisms. These have seeped into the academic literature, making it difficult to separate fact from opinion or fiction. The more these claims are recited as fact, the more they mask the flaws, failings and risks of innovative financing mechanisms while contributing to an ill-informed union between innovative finance and PPPR (what network analysis calls ‘citation inertia’). Claims of success and achievements reviewed in this article, be they in terms of lives saved, children vaccinated or vaccines developed at speed, are so pervasive that they have almost achieved a taken-for-granted status, whilst permeating the scholarly literature. This has been enabled by the scarcity of independent evaluation to either support or refute these claims.

However, the actual impact of these instruments on disease is difficult to assess in the absence of such independent evaluation. To illustrate this point, WHO Director-General, Dr Tedros, welcomed the conditional marketing authorisation of Ervebo, claiming the “vaccine has already saved many lives in the current Ebola outbreak, and the decision by European regulator will help it to eventually save many more” [132]. However, the 2014 West African outbreak is the only Ebola outbreak in history to kill more than 10,000 people, while the vaccine provides only imperfect protection against infection and death, as shown in an observational study from the DRC outbreak that reports a case fatality rate of 25% among the vaccinated [133]. This shows that claims of ‘many lives’ or ‘countless lives’ saved through the use of the vaccine, developed with innovative financing, are highly questionable. Such claims must be objectively weighed against disease burden or evidence of the effectiveness of funded countermeasures. The same applies to estimates cited by Gavi that COVAX helped save at least 2.7 million lives. This is (if following other examples) based on modeling and addresses only lives predicted to be lost from Covid-19, not all-cause mortality affected by direct and indirect (opportunity) costs, which would be necessary to include to know whether the mechanism was successful from a broad public health viewpoint.

Lives saved must also be weighed against those that could have been saved from other higher-burden health threats had those funds been diverted there instead. Further, those financing mechanisms that borrow from the future through instruments such as the IFFIm and the sale of bonds must demonstrably bring gains to future populations greater than the costs that are being foisted onto them in bond payouts. This is problematic, as we cannot well predict future illness – who fifty years ago would have predicted the current lowering of North American life expectancy due to metabolic disease? That is to say, while such mechanisms mobilize funds, they also push the financial risk to the future within which competition for funds for competing health priorities is unknown. Bearing this in mind would mitigate against use for diseases that occur sporadically in widely-dispersed populations in outbreaks of generally short duration (Ebola) or arise due to mutations that are unrecognized by prior (vaccine or naturally-induced) immunity, such as influenza, unless major reductions in otherwise unavoidable economic costs could be demonstrated. Otherwise, we are borrowing from the future to fund a current problem.

### Overstated potential to mobilise funds for PPPR

The WEF suggests that the potential of innovative financing mechanisms makes vital investment in pandemic prevention “more palatable by helping to spread the cost” [4]. This is premised on the logic that “the $12.5 trillion cost of COVID to the global economy, [taught] governments… that if they don’t invest the billions of dollars needed to achieve global pandemic preparedness, then they will surely pay for it later” [4]. This claim rests on the assumptions that innovative financing has the potential to mobilise the billions of dollars needed for PPPR, that such measures will prove effective in a future outbreak, and that the massive costs of the Covid-19 event were not otherwise avoidable [48]. Assuming that this US$10.5 billion per annum estimate by the WHO, World Bank and G20 to finance PPPR is correct (in which there are doubts) [134], it is worth putting the amounts historically generated by innovative financing mechanisms into perspective.

With regards to mobilising funding, the generation of revenues was cited as a success story for all innovative financing models reviewed in this article. The amounts generated by the mechanisms reviewed here are as follows, from low- to high-end: the PEF provided US$500 million (through a combination of bonds and derivatives), the Gavi Ebola APC raised US$5.8 billion, the IFFIm mobilised US$9.7 billion and the Gavi COVAX AMC raised US$12 billion. The total amount raised by all these mechanisms to date is US$28 billion, which in theory would fall short of covering three years of estimated PPPR ODA funding needs. Focusing on the mechanisms closest to the US$10.5 billion PPPR financing need, it took the IFFIm roughly 17 years (from its launch in 2006 to March 2023) to raise US$9.7 billion [38], while the COVAX AMC managed to raise only US$3.43 billion per year (a total of US$12 billion over 3.5 years, from June 2020 to its closure in December 2023) [110]. While going into further detail is beyond the scope of this article, the funding generated by major PPPR financing mechanisms so far (taken together or apart) is nowhere near the US$10.5 billion per annum ODA PPPR financing estimate provided by the WHO, World Bank and G20. This highlights the limited contribution prospective innovative financing tools could make to raising funds at the scale suggested for PPPR. While this is to be expected with innovative financing instruments, intended to supplement traditional sources of health financing, it appears to be overlooked in various statements about the “huge” potential of these mechanisms for PPPR in the grey literature [4]. While one may argue that it is not impossible to raise higher amounts through innovative financing in the future, it is highly improbable based on their past performance and poor track record of meeting their own targets. In this case, it is important to understand that innovative financing has promise to be a small part of the solution (if designed properly), but is very unlikely to be the panacea some commentators seemingly suggest (most likely for advocacy reasons to foster greater investment).

In this line of thought, this review of innovative financing mechanisms has also demonstrated that raising money alone is not sufficient, it then requires swift, efficient and equitable distribution. The PEF is notorious for its failure to pay out on two occasions in the face of Ebola outbreaks in the Democratic Republic of Congo [135]. In addition, it came under fire for the time it took to trigger payouts in the context of the Covid-19 pandemic [136] and how “tiny [it was] in comparison to what [was] needed” [60], with just US$195.84 million payout announced three months after a PHEIC was announced [137]. Among other flaws, the PEF’s failings can largely be attributed to its high disbursement threshold criteria and “the length of time that needs to elapse before the [payout] criteria are even assessed” [60]. However, these issues are not limited to the PEF.

Hughes-McLure and Mawdsley (2022) identify a concerning pattern in the operation of the IFFIm up to the first half of 2021 (the period covered by their analysis), whereby the significant sums raised by the mechanism did not translate into significant sums reaching Gavi. Even though larger sums were eventually disbursed in the context of the Covid-19 pandemic, Hughes-McLure and Mawdsley suggest that this did not happen in a timely manner. Disbursement caveats in innovative financing mechanisms, which can make payouts “too expensive, too slow and too small,” as exhibited in the operation of the PEF and the first 15 years of the operation of the IFFIm [138], appear to fit the requirements of a pandemic response poorly.

### Lack of transparency and accountability, conflicts of interest and excessive private sector profiteering

A lack of transparency found in aspects of all the innovative financing mechanisms assessed in this review should also temper enthusiasm for their continued application. The response to Covid-19 was marked by a ‘transparency problem’ from the get-go, which extended to the Gavi COVAX AMC, criticised for the lack of transparency surrounding its ultimate cost and inner workings. With a governance structure that allowed its richest members to strike deals with vaccine manufacturers and procure vaccines outside of COVAX as well as to “cherry-pick” products from its portfolio, while making its poorest members dependent on aid, COVAX catered to the interests of highest bidders and manufacturers, enabling the latter to prioritise supply to their high-paying clients [119]. Similarly to COVAX, the Gavi Ebola APC was censured for a lack of transparency around funding contributions, R&D funding, the pricing structure of the Ervebo vaccine, including development incentives, and the specific terms of the Gavi-Merck agreement. Such opacity creates favourable conditions for conflicts of interest with private sector actors profiting at the cost of donors and intended beneficiaries.

Furthermore, innovative financing mechanisms have proved to be a bad deal for donors and excessively beneficial for the private sector. The Gavi Ebola APC included no financial or other repercussions for Merck failing to develop the Ervebo vaccine within the agreed timeline. Gavi was unable to recoup their investment even though Merck failed to hold up their end of the deal. The APC agreements that de-risked pharmaceutical companies’ investment in developing vaccines for Covid-19 suffered from similar issues, including the omission of provisions for supply disruptions and guaranteeing indemnification against liability by the buyers, allowing vaccine manufacturers to evade accountability [117].

Evidence of significant profit-making at the expense of beneficiaries and donors in the functioning of the IFFIm put forward by Hughes-McLure and Mawdsley (2022) is yet another warning of what might be understood as excessive profiteering by private sector actors, which comes at the cost of donors and beneficiaries, and a high opportunity cost that should be taken into account when considering the use of innovative financing frontloading mechanisms to fund PPPR. In other words, it is understandable that a level of profit is required to incentivise private sector investment, and that some level of risk must be absorbed to stimulate the model. Yet, there is a difference between reasonable profits as incentive and disproportionate profit at the expense of beneficiaries. As a result, there is a clear need to strike an appropriate balance, a balance that is currently not practiced.

In terms of expense, the authors suggest that the opaque financial mechanisms through which large sums are transferred to private sector actors make the IFFIm an expensive model that comes at a significant cost for donor governments, Gavi and its beneficiaries. Hughes-McLure and Mawdsley’s observation that there is a mismatch between the significant sums of government aid and comparable bond issuances, and the limited funds received by Gavi, raises concerns about opportunity costs associated with the use of this mechanism.

While the private financial actors are justifiably drawn to bonds that represent low-risk source of significant windfall, the IFFIm is far from a bonanza for donors and beneficiaries that bear the disproportionately high costs and risks to secure the rewards reaped by their private sector counterparts. Bonds like the IFFIm have significant implications for donor governments, because they require them to commit to legally binding long-term conditions, assuming all of the risk while intellectual ownership and profits from vaccine discovery and manufacturing accrue to pharmaceutical partners. This raises concerns regarding policy adaptability and national interest, since commitments can last up to 29 years, locking governments into financial commitments regardless of efficacy and changing health, economic and social contexts. The concentration of decision-making in the Global North and the lack of inclusivity regarding national and regional interests raises serious concerns about policy legitimacy, contextual suitability, national ownership, and effectiveness. It ignores normative ambitions in global health policy to promote equity, decolonialization and self-sufficiency; issues that were centre stage in recent negotiations on the Pandemic Agreement. The uncertainty surrounding the IFFIm’s contribution to global health (due to the lack of independent assessment), coupled with the financial and political costs of the model and concomitant opportunity cost, warrant paying closer attention to the hidden mechanisms through which money is transferred from the public to the private sector, “the mechanisms at work distributing risk and the ways aid from governments is used to mitigate risk for private capital” [14].

### Opportunity costs and the potential for resource diversion away from diseases of greater burden

Reflections on opportunity costs with the use of innovative financing mechanisms for PPPR raise questions regarding their efficiency (i.e., the lack thereof) in using funding in a way that benefits all donors, rather than exclusively the private sector. This potential liability was evident in the Gavi Ebola APC, where the materialisation of the risk of overbuying and overpaying to tackle a relatively small disease burden amounted to a waste of critical global health financing. This undoubtedly diverted vital financial resources from diseases of higher burden. Similar potential opportunity costs exist in the context of PPPR that must be factored into financing considerations.

### Tendency to focus on discrete areas of PPPR

The application of innovative financing mechanisms in the context of PPPR indicates that these instruments tend to be largely response models created to handle surges for an already active outbreak (Ebola and Covid-19), rather than addressing an intent to prepare for them. Yet, even when focused on response, failures quickly emerge. For example, the PEF surge fund has illustrated the importance of tackling complicated payout criteria and disbursement issues when designing PPPR financing tools. Doing so properly would require equally complicated indicator / target models with costly monitoring and evaluation mechanisms to appraise programmes. It is hard to imagine how bonds, market commitments or a loan system could achieve this equitably. This suggests a need for research to assess whether a grant-based system would be more viable. However, this could necessitate an even narrower focus (if based on common results-based models), thus threatening to create disjointed siloes of PPPR excellence at the expense of comprehensive public health approaches.

A related tendency revealed in innovative tools to finance PPPR is its historical overemphasis on mass vaccination as the principal form of outbreak response [128, 139]. This was reflected in Covid-19 response strategies, but also worryingly mirrors recommendations within new PPPR policies such as “100 Days to Vaccine,” the new International Pathogen Surveillance Network and the global Medical Countermeasures Platform. As a result, a fetishization of vaccines within innovative finance threatens to crowd out more broadly-impacting PPPR investments such as health system strengthening and upstream social determinates including the promotion of healthy lifestyles [128].

The overreliance on vaccine strategies is not surprising. With the notable exception of the PEF, the major innovative financing mechanisms reviewed here have been spearheaded and managed by Gavi to fund its own programmes and initiatives. To avoid this narrow focus, which is ultimately insufficient for a holistic approach to financing PPPR [139], future attempts at utilising financing models for PPPR should look beyond Gavi to lead the endeavour and ideally at a partnership of diverse global health stakeholders, including WHO Member States from the Global South. This would not only contribute to a more comprehensive public health approach to PPPR at the heart of proposed innovative financing solutions but maximise the chance of more equitable representation of the interests of beneficiaries.

### Proposed measures and alternative approaches to address the shortfalls of innovative financing mechanisms for PPPR

In addition to raising awareness of where innovative financing mechanisms for PPPR have fallen short in the past, innovative financing scholars have offered guidelines and solutions to address these shortfalls. Some suggest improving the design of existing innovative financing models or measures to mitigate the shortfalls. Thornton et al. propose mechanisms to reduce the risk of overbuying and the resulting negative impact of APCs on equality of access, including making “greater use of pooling and resale markets” rather than purchase commitments, a “pandemic treaty governing access to necessary medical countermeasures,” or relying more on AMCs rather than APCs [17]. Towse et al. propose a type of AMC – Benefit-Based Advance Market Commitment (BBAMC) – to incentivise the development of, and ensure equitable access to, second- and third-generation pandemic vaccines in the context of Covid-19 [20]. Others, go beyond innovative models to offer entirely different solutions to the issues innovative financing models have sought to address. Instead of relying on IFFIM-like frontloading mechanisms, Hughes-McLure and Mawdsley offer an alternative approach to solving the problem of vaccine access for children in LMICs, which calls for solutions addressing the root causes of the issue rather than looking to close the investment gap through “funds intermediated through capital markets” [14]. These solutions include “reforming intellectual property right law” to tackle inflated pharmaceutical prices, direct payments or grant funding for health centres and medical staff training to improve healthcare system infrastructure, “tackling capital flight” from LMICs, and reforming the global tax system to mitigate the impact of LMICs inability to raise sufficient taxes [14].

Given the unprecedented demand to raise funds for PPPR, adding an additionality component for private sector partners into the models examined here would increase the amount of funds raised for these initiatives while also helping to address concerns that donors shoulder all the risk. For example, these models could require a 10-cent co-investment from private sector partners for every US$1 investment from donors. This could generate three effects: 1. Immediately increase the amount of available funds; 2. Spread investment risk to include all partners while not fully undermining profit incentives needed to mobilise the private sector; 3. Make private sector actors more accountable by linking return on investment strategies to include all scheme stakeholders / shareholders. However, this could reduce the attractiveness of the mechanism for key investors, with potentially mechanism-ending effects. As a result, pursuing this alternative would require careful assessment of feasibility restraints before wholesale adoption.

Engaging the private sector is only part of the challenge. To address the PPPR financing gap, we also need to figure out how to make innovative “all-for-one-and-one-for-all approach[es] to defeating the pandemic,” like the Gavi COVAX AMC, work in the real world [115]. As Gavin Yamey, who was part of a working group that discussed COVAX’s design in 2020, suggests, the concessions that were made to incentivise HICs to join COVAX are indicative of the extent of the problem [115]. According to Yamey, that would require either stronger incentives or a mandatory-participation mechanism for all nations, making this challenge “a difficult nut to crack” [115]. The challenge is exacerbated by the need to mobilise funding at speed, which hampered ACT-A’s response to Covid-19, according to Open Consultants’ evaluation of the initiative [114]. In response to this challenge, they suggest ensuring that contingent funding is “available on day zero of the next pandemic” through a “pandemic Advance Commitment Facility with access to a credit line [to] help… secure orders earlier to promote a faster and more equitable global response” than during the last pandemic [114].

## Conclusion

The use of innovative financing mechanisms for PPPR is not a novel proposition. On the contrary, it has been wielded to manage infectious disease outbreaks for a decade. The late response and limited national capacity to respond to the 2014 Ebola outbreak created an impetus to apply innovative financing mechanisms to PPPR. This led to the creation of the PEF and the Gavi Ebola APC, with use of IFFIm funds to support the latter. More recently, we have witnessed a proliferation of such mechanisms in the context of the Covid-19 pandemic – an AMC and multiple APCs, alongside the continued use of the IFFIm and the PEF. With the exception of the PEF, these mechanisms have been lauded by their champions as milestones in public health preparedness and response.

The Covid-19 experience also supercharged the conversation around the use of these mechanisms in the future, albeit led mostly by Gavi, IFFIm and their affiliates. These entities appear to have drowned out the few critical voices in the innovative financing space and there is a dearth of independent evaluation and evidence. This article sought to redress the balance by closely examining existing claims to, and cited evidence of, the effectiveness of these mechanisms for PPPR. The intent was to present an informed argument about their future use within the first comprehensive overview of innovative financing tools for PPPR.

To borrow a phrase from Ritchie and Plant’s analysis of the PEF, innovative financing mechanisms have at best been “good ideas executed badly”, [60] be it due to design flaws, trade-offs and risks, commitments to bad deals for donors and beneficiaries, untimely and insufficient disbursements, or lack of transparency and accountability. The result is that despite some successes in mobilising resources for PPPR interventions, encouraging demand and providing market-enhancing incentives for medical countermeasures, they have rarely delivered their full promise. History tells us that innovative financing mechanisms bring a degree of uncertainty as to how donor funding is spent, which leads to questions about whether these mechanisms deliver the ‘value for money’ they claim to embody. There is clear evidence across the four mechanisms analysed here that none of them have fully lived up to their promises and claims to effectiveness, suggesting that self-evaluations mostly focus on advocacy to market new investments rather than a serious effort to present objective evidence of performance. Put simply, the innovative financing toolkit contains a range of instruments that don’t do what it says on the box. This reality has been obscured by a proliferation of anecdotal claims by entities and actors involved. Such anecdotes about the success of the innovative financing mechanisms reviewed have been spared independent evaluation, including of their impact on disease burdens. The absence of counter-verification of the source material raises questions about the validity of these claims and whether they have created a false perception of impact.

This article has exposed a multitude of issues with the historical and prospective use of these mechanisms, raising questions as to their effectiveness for PPPR and beyond. Further research is needed to evaluate their impact, taking into account the relative disease burden and the significant risks and opportunity costs arising from their application. As a first step, these mechanisms require independent evaluation – or evaluation from government underwriters who assume the greatest financial risk – to identify and confirm actual performance and untapped potential (there have been some external evaluations, such as those conducted by the United Kingdom and Norway, although these are exceptions and not the norm, while also not fully addressing potential conflicts of interest, given country / industry investment interests). It cannot be assumed that simply more investment is better, since the likely result will be more of the same. Moreover, analyses should include a cost–benefit analysis with realistic return-on-investment calculations that can better justify the risk versus reward. Otherwise, it remains unclear what the overall results of these investments have been, or potentially will be, leading to speculative investment with its associated opportunity cost. Ideally, this should be supplemented with a realist evaluation to better understand why certain mechanisms work better than others and for whom [140]. Although commissioning this evidence base will require time and resources, global health would be better served by aligning policy with robust evidence. Such analysis should be undertaken prior to further large-scale innovative financing investments to try and resolve complex PPPR issues.

Furthermore, it should also be born in mind that past funding achieved through these mechanisms pales in comparison to the financing estimates for PPPR. In other words, innovative financing is not a panacea and the devil’s in the detail. Contrary to the hype, innovative financing mechanisms have tended to benefit private interests over their intended beneficiaries and appear incapable of addressing the unprecedented public health budget being proposed for PPPR. The size of the proposed funding for PPPR, the associated opportunity cost and concomitant risk of diverting resources from diseases of higher burden mean that if we are to fund PPPR, we first need to review both the direction and the mode of travel.

## Data Availability

No datasets were generated or analysed during the current study.

## Appendix

**Table 1:** Summary Table of Innovative Financing Mechanisms for PPPR

Funding mechanism	International Finance Facility for Immunization (IFFIm)	PEF/PEF 2.0	Gavi Ebola Advance Purchase Commitment (APC)	Gavi COVAX Advance Market Commitment (AMC)
**Year**	2006	2016/closed 2021	2016	2020/closed 2023
**Design**	An innovative financing mechanism employing ‘frontloading’—an approach, in which vaccine bonds backed by government guarantees (giving them a good rate of return), are issued on global capital markets. When purchased, these long-term donor pledges are converted into immediately available funds for global health programs. Proceeds from IFFIm vaccine bond issuances allow Gavi to purchase larger volumes of vaccines for child immunisation.	An innovative insurance-based financing mechanism, comprising two independent, complementary windows – a cash window and an insurance window, through which the financing could be provided: ➢The cash window could provide fast financial support to eligible countries fighting disease outbreaks, outside the coverage of the insurance window. ➢The insurance window (associated with pandemic bonds) could provide payments over a three-year period to a maximum of US$425 million for all qualifying outbreaks.	A forward-looking binding bilateral contracts whereby one party agrees to purchase a fixed volume of a qualifying product from a specific manufacturer / supplier at a pre-agreed price if / when certain conditions are met.	A forward-looking binding contract that guarantees a market for a not-yet-available product, whereby one party (a buyer / donor government) commits to purchase a fixed quantity of a product (in development) at a pre-agreed price or to subsidise the purchase of said product by a third party (low-income country) from any manufacturer / supplier that would be able to produce a qualifying product.
**Presumed value and benefits**	***Overall:*** Fast-tracking the availability of predictable, long-term funds for health interventions / programs and medical countermeasures ***For donor governments:*** Allows them to have an immediate impact on saving lives by putting pledges to work immediately while offering a cost-effective way to spreading contributions over a longer period ***For investors:*** Offers attractive fixed returns, opportunity to diversify their portfolios while aligning them with a social cause ***For Gavi:*** - Increasing the availability and predictability of funding for Gavi’s vaccination programmes and initiatives, allowing it to use funds flexibly when most needed (over a shorter or longer period) - Ensuring long-term predictability of funding helps Gavi drive down vaccine prices and secure supply ***For LMICs:*** Could accelerate the delivery of life-saving vaccines ***For PPPR:*** - Could improve global pandemic preparedness faster through fast-tracking funding for health and medical interventions, while allowing donor governments to spread the cost over future years - Vaccine bonds can enhance capacity and efficiency in the provision of healthcare services and maintain them, thus accelerating the pace of economic recovery for pandemic-stricken sectors	***Overall:*** To provide surge financing through its insurance window for response efforts to a large-scale outbreak of a disease on the WHO Priority Disease List, affecting the world’s poorest countries, from reaching pandemic proportions	***Overall:*** Incentivises R&D and production capacity for a product before firm demand materialises ***For manufacturers***: Helps mitigate the unusually high degree of demand uncertainty created by pandemic events ***For buyers / HMICs:*** Helps mitigate supply risk and secure priority access to a product in high demand ***For LMICs:*** Creates demand / market for, and promotes access to, in-demand health products they lack the means to develop or purchase ***For PPPR:*** Helps mitigate the unusually high degree of demand uncertainty created by pandemic events and secure the availability and supply of medical countermeasures (e.g. pandemic vaccines) in the face of (anticipated) increasing demand	***Overall:*** Incentivises R&D and production capacity for a product before firm demand materialises ***For manufacturers:*** Creates a subsidised market for health products and insulates them from aggregate demand risk ***For buyers / HMICs:*** Helps mitigate supply risk and secures priority access to a large pool of products in high demand ***For LMICs:*** Creates demand / markets for, and promotes access to, in-demand health products they lack the means to develop or purchase ***For PPPR:*** Helps mitigate the unusually high degree of demand uncertainty created by pandemic events and secure the availability and supply of medical countermeasures (e.g. pandemic vaccines) in the face of (anticipated) increasing demand
**Purpose / rationale for establishing of example mechanism**	To raise funds for the achievement of the Millenium Development Goals and fill an estimated funding gap of $30-$70 billion annually until 2015. Originally intended to support Gavi’s childhood immunisation programmes, the IFFIm has since expanded its remit to mobilise funding for Ebola response, CEPI, COVAX and pandemic prevention.	Emerged in response to the challenge of rapid funding mobilisation from the international community to contain a pandemic outbreak, brought to the fore by the 2014 Ebola crisis in West Africa.	To protect against future Ebola outbreaks by supporting the provision of investigational Ebola vaccine doses (developed by Merck), and when fully licenced, to make it available to the poorest countries in the world at the lowest possible price.	To ensure access to COVID-19 vaccines by supporting the participation of 92 lower-income economies in the COVAX Facility.
**Purported achievements of example mechanism**	➢Mobilised US$9.7 billion over 18 years for Gavi’s immunization programmes ➢Provided US$5.8 billion to Gavi – 18% of its programme budget (up to 31 December 2023) ➢Supported more than 13 vaccine introductions ➢Helped Gavi vaccinate over 1B children, save 17 million lives, and reduce child mortality by half across 73 LICs ➢Making funds available to Gavi sooner through frontloading has contributed to saving more lives than would have been possible under traditional rounds of donor pledging ➢Mobilising US$975 million for PPPR through the first social Covid-19 bonds and disbursing over US$247.9 million to the Gavi COVAX AMC up to 31 December 2023 ➢Covid-19 vaccine bonds allowed donors to fast-track funding and facilitate ‘equitable’ global access	➢The *cash window* paid out US$61.4 million to fight two Ebola outbreaks in the Democratic Republic of Congo (DRC) in 2018 and 2019 ➢The *insurance window* was triggered on 27 April 2020 and US$195.84 million were allocated to 64 of the world’s poorest countries with reported cases of Covid-19	➢The APC incentivised manufacturers to speed up vaccine development ➢350,000 people were vaccinated on a compassionate use basis in Guinea and in the 2018–2020 outbreaks in the Democratic Republic of Congo (DRC) ➢The APC was a first-of-its-kind agreement between Gavi and the vaccine manufacturer, which set a precedent for fast-tracking development and production of vaccines against Covid-19	➢Helped build “the largest portfolio in the world” of “11 vaccine candidates across four technology platforms (of which 10 received regulatory approval).” ➢US$12 billion in donor funding for the AMC, allowing the latter to ship over 2 billion doses of the Covid-19 vaccine and safe injection devices to 146 economies ➢Estimated to have averted more than 2.7 million deaths in AMC countries ➢COVAX supplied 74% of LMICs Covid-19 vaccine doses during the pandemic ➢Covid-19 vaccine rollout in HMICs has been the fastest in history
**Shortcomings**	➢The impact of frontloading in terms of lives saved and claims to saving more lives through fast-tracking funding are poorly substantiated ➢No evidence of aid additionality – the IFFIm channels Gavi funds through private markets rather than raising additional funding ➢Evidence of an expensive model that comes with significant financial, political and opportunity costs ➢Locking governments into financial commitments regardless of efficacy and changing health, economic and social contexts ➢Potential for excessive private sector profiteering at the expense of public donors and beneficiaries ➢Control concentrated in the Global North ➢Lack of accountability and insufficient independent scrutiny of mechanism	➢Bad design led to failure to pay out the earmarked capital investment / surge funding to address outbreaks three times (the 2018 Ebola outbreak, the 2019 Ebola outbreak, and the Covid-19 outbreak – where too little funding to the world’s poorest countries came too late). This was due to the bond criteria, requiring the disease to spread across national borders before affected countries can receive the payout ➢Deemed financially inefficient, costing more than it brought in ➢Perceived as a good deal for investors but a bad deal for global health, as it allowed for private profiteering at the cost of development and health ➢The type of surge funding provided by the PEF is unsuitable for a holistic public health approach to PPPR financing, as it can only be used to finance outbreak / pandemic response, while ignoring pandemic preparedness and prevention ➢Its maximum coverage falls short of PPPR financing needs	➢Risk of overbuying and overpaying when demand does not materialise ➢A lack of transparency around price-setting, funding contributions, R&D funding, development incentives and pricing structure of the Ervebo vaccine ➢Manufacturers not held to account for vaccine development, licencing and procurement delays ➢Limited and rapidly contained Ebola outbreaks since 2021 (and a historical pattern of Ebola outbreaks resolved through non-vaccine interventions) raise questions about cost-effectiveness and justification of large investment into rapid vaccine development, especially given its inevitable opportunity costs on other interventions ➢Extensive use of APAs by HICs contributes to inequity in access to vaccines, as HICs tie up supply, especially in the early stages of an outbreak	➢Despite the historic achievement of the fastest rollout of vaccines in HMICs, LMICs received vaccines much later ➢The failure of supply to meet demand (especially during 2021) and the inequitable distribution of vaccines between countries at different income levels might have cost thousands of lives ➢Vaccine nationalism limited COVAX’s ability to raise funds from affluent donors and meet its procurement goals for vulnerable populations ➢The self-financing arm of the COVAX Facility was considered a failure, as HICs hardly used it due to securing vaccines through bilateral deals outside COVAX ➢Manufacturers prioritising supply to high-paying clients contributed to inequitable access to vaccines ➢Preferential arrangements offered to HICs to incentivise participation in COVAX undermined the principle of equal treatment between self-financing participants and AMC countries ➢Mechanism was built on an assumption that the appropriate response to Covid-19 requires vaccinating ‘all’, which is questionable from a public health perspective ➢The COVAX AMC model allows for excessive private sector profiteering at the cost of donors and beneficiaries and comes with potentially high opportunity costs ➢Issues of transparency and accountability raise concerns about conflict of interest, profiteering, and blurring public and private goods

## Footnotes

1 It is worth noting that this is specifically not the case for a scattered outbreak disease such as Ebola (or for one like Flu, where outbreaks result from genetic shifts).

2 Methodology for critical financial analysis being developed by Hughes-McLure (unpublished).
